# Circulating microRNAs as potential candidate biomarkers of left ventricular reverse remodeling after myocardial infarction: A pilot study

**DOI:** 10.1371/journal.pone.0360414

**Published:** 2026-10-15

**Authors:** Roksana Gozdowska, Marcin Konopka, Anna Hojka-Osińska, Grzegorz Jas, Katarzyna Czarzasta, Agnieszka Cudnoch-Jędrzejewska, Wojciech Braksator, Dominika Klimczak-Tomaniak, Marek Kuch

**Affiliations:** 1 Department of Cardiology, Hypertension and Internal Diseases, Mazovian Brodnowski Hospital, Medical University of Warsaw, Warsaw, Poland; 2 Department of Sports Cardiology and Noninvasive Cardiovascular Imaging, Faculty of Medicine, Medical University of Warsaw, Warsaw, Poland; 3 Bioinformatics Facility, International Institute of Molecular and Cell Biology, Poland; 4 Department of Experimental and Clinical Physiology, Laboratory of Centre for Preclinical Research, Medical University of Warsaw, Warsaw, Poland; 5 Department of Heart Failure and Cardiac Rehabilitation, Medical University of Warsaw, Warsaw, Poland; Isfahan University of Medical Sciences, IRAN, ISLAMIC REPUBLIC OF

## Abstract

**Background:**

Myocardial infarction (MI) induces cardiac remodeling and lead to heart failure. Some patients experience reverse remodeling, characterized by improved LV function. MicroRNAs (miRNAs) regulate gene expression and play a crucial role in remodeling processes.

**Objectives:**

We aimed to investigate the role of miRNAs in cardiac reverse remodeling after MI and identify candidate miRNAs that could predict better response to cardiac rehabilitation.

**Methods:**

The study enrolled 39 patients following their first MI. For final analysis, 10 patients meeting echocardiographic criteria for LVRR at 8-week follow-up (absolute LVEF increase ≥ 10% or LVESV reduction ≥ 15%) were matched with 10 patients with stable LV status (SLVS). All patients completed a cardiac rehabilitation program. Expression of 375 circulating miRNAs was quantified – those with higher baseline accumulation in the LVRR group were identified as candidate biomarkers and their target genes were analyzed.

**Results:**

Twenty patients (mean age 57 ± 9 years) were analyzed. At baseline, the SLVS group exhibited significantly higher LVEF compared to the LVRR group (53% vs. 44%, p = 0.013). Baseline regional contractility impairment in all LVRR patients was reversed in 70% of them at follow-up. Fourteen miRNAs (-15b-5p, −24-3p, -29c-3p, -99b-5p, −127-3p, -146a-5p, -199a-3p, −361-5p, −502-5p, −625-5p, -376c-3p, -18b-5p, −342-5p, and −429) were identified as candidate LVRR biomarkers. Enrichment analysis linked their targets to PI3K-Akt, MAPK, FoxO, and HIF-1 pathways.

**Conclusions:**

This study identifies 14 candidate miRNAs as LVRR biomarkers post-MI. These findings highlight the biological relevance of non-coding RNA and support the need for further research and validation in large, non-pooled cohorts.

**Unstructured abstract:**

Myocardial infarction triggers adverse cardiac remodeling, though some patients achieve left ventricular reverse remodeling (LVRR), marked by functional and structural recovery. This pilot study investigated 39 patients to identify circulating microRNAs (miRNAs) that predict positive responses to cardiac rehabilitation. Comparing 10 LVRR responders against 10 stable patients, we quantified 375 miRNAs via RT-qPCR. At baseline, the stable group had higher ejection fractions (53% vs. 44%, p = 0.013), but 70% of LVRR patients reversed regional contractility impairment by the 8-week follow-up. We identified 14 potential biomarkers, including novel candidates (miR-502-5p, miR-376c-3p, and miR-18b-5p), whose target genes enrich pathways like PI3K-Akt, MAPK, and FoxO. These findings bridge the gap between preclinical data and clinical settings, highlighting the therapeutic potential of non-coding RNAs in cardiac reverse remodeling and necessitating further validation in larger cohorts.

## Introduction

Myocardial infarction (MI) is a major cardiovascular event that causes loss of cardiomyocytes and triggers a sequence of structural and functional changes in the heart, defined as adverse remodeling [1]. A key aspect to developing effective therapeutic strategies to prevent the progression of post-MI heart failure is understanding the cellular mechanisms underlying cardiac remodeling, including inflammation, fibrosis and hypertrophy. After MI, the heart can partially recover its shape and function with appropriate medical treatment and interventions, improving the outcomes and quality of life of those affected.

Reverse remodeling (RR) after MI refers to structural and functional improvement in myocardial function, characterized by reduced left ventricular (LV) dilatation and improved LV ejection fraction [2]. This phenomenon is observed in a significant subgroup of post-MI patients, although its incidence varies depending on the therapeutic interventions and individual patients’ characteristics. Previous studies estimate that approximately 10–40% of patients with HF show RR within one year of optimized therapy [3,4]. Parameters of reverse remodeling can be evaluated by imaging studies, most commonly echocardiography, which remains the most accessible, non-invasive examination.

An important component in promoting the recovery of cardiac function after myocardial infarction is cardiac rehabilitation (CR). It is a complex intervention that includes strength training, aerobic exercise, health education, psychological consultation, and lifestyle modification. The effectiveness of CR has been widely described, existing literature demonstrates that it reduces the risk of reinfarction and heart failure [5], lowers cardiovascular mortality [6] and even all-cause mortality [7–9].

Post-myocardial infarction cardiac remodeling is a highly orchestrated process that requires crosstalk across the cellular and extracellular environment. Recent studies investigating the molecular mechanisms that may enhance cardiac reverse remodeling have highlighted the potential of microRNAs, as they modulate key biological pathways involved in cardiac remodeling [10,11].

MiRNAs are small non-coding RNAs that regulate gene expression by binding to target mRNA sequences. Their involvement in key cellular processes – including proliferation, differentiation and apoptosis – makes them potent regulators of cellular behavior in damaged heart tissue [12].

While microRNAs are central to mediating this intercellular communication, they do not operate in molecular isolation. Instead, they function within broader, highly complex epigenetic networks, including interactions with extracellular vesicles and long non-coding RNAs (lncRNAs). For instance, recent comprehensive review highlights how the broader non-coding RNA interactome—such as regulatory axes involving lncRNAs like MALAT1—modulates critical post-infarction responses, including apoptosis, inflammation, and angiogenesis [13]. Recognizing this vast cellular and extracellular cooperation provides an essential biological context for identifying specific circulating miRNAs as predictive biomarkers of left ventricular recovery. Such biomarkers would provide valuable insights into the reverse remodeling process support the development of targeted therapeutic strategies aimed at improving clinical outcomes [14].

In the present study, using a comprehensive microRNA assay panel, we assessed the expression of 375 miRNAs in patients following myocardial infarction, who received optimal medical therapy and subsequently underwent cardiac rehabilitation. We compared miRNA profiles between patients who exhibited LV reverse remodeling, as improved echocardiographic parameters at 8-week follow up, and those whose cardiac function remained unchanged relative to baseline assessment performed during hospitalization. Among the detected miRNAs, we identified those with high potential to serve as biomarkers of cardiac reverse remodeling and subsequently analyzed their predicted target genes.

In the next step, we analyzed the predicted target genes of the miRNA biomarker candidates and identified the signaling pathways associated with these genes. For the key enriched pathways, we constructed a gene linkage network to illustrate the relationships between pathways and the genes most strongly associated with our miRNA candidates. This approach enabled a deeper understanding of how these miRNAs may influence remodeling processes and the potential mechanisms underlying cardiac reverse remodeling.

## Materials and methods

### Study design

We conducted a pilot study to identify candidate miRNAs that are potential prognostic biomarkers in cardiac reverse remodeling post-MI and assess their target genes and the pathways they modulate. This study was approved by the local institutional ethics committee (Medical University of Warsaw Bioethical Committee, approval number KB/147/2022). A written informed consent was obtained from each patient before inclusion. All procedures were conducted according to the guidelines of the 1964 Declaration of Helsinki and its later amendments.

Thirty-nine consecutive patients who experienced the first occurrence of MI within the period from 28.08.2023 to 08.02.2024 at Mazovian Brodnowski Hospital in Warsaw, Poland were recruited. The diagnosis of MI was based on the European Society of Cardiology Guidelines [15]. All patients received direct percutaneous coronary intervention (PCI). Included patients were required to have successful reperfusion and no chronic total occlusion (CTO) of any of the coronary arteries. We also excluded patients with history of previous MI, heart valve disease, pulmonary embolism, atrial fibrillation, severe renal or hepatic dysfunction, acute inflammation or neoplastic process (Table 1.). Standard secondary prevention and treatment of coronary heart disease were administered in all patients according to evidence-based optimal medical therapy (OMT) after acute myocardial infarction [15].

**Table 1:** Inclusion and exclusion criteria. eGFR- estimated glomerular filtration rate; NSTEMI- non-ST-elevation myocardial infarction; STEMI- ST-elevation myocardial infarction.

Inclusion criteria:	Exclusion criteria:
First occurrence of acute STEMI or NSTEMI	History of prior myocardial ischemia
Age > 18 years	Atrial fibrillation
Informed consent	Concomitant diseases • chronic kidney disease (eGFR < 30 ml/min/1.73 m2) • acute or chronic liver disease (Child-Pugh B or C) • chronic lung disease, pulmonary hypertension • active neoplasm
	Severe ventricular arrhythmias, atrio-ventricular block grade II or III
	Moderate or severe valvular disease
	Pericarditis or myocarditis

### Study protocol

On day 3 after MI (during hospital stay) patients were screened for inclusion to the study, clinical examination was performed, blood sample for miRNAs analysis was collected. Baseline echocardiographic examination included evaluation of left ventricle end-systolic volume (LVESV), left ventricle end-diastolic volume (LVEDV), biplane ejection fraction (EF) using Simpson’s method, global longitudinal strain (GLS) and peak atrial longitudinal strain (PALS) measurements using speckle tracking.

After discharge all patients in this study underwent a cardiac rehabilitation program consisting of controlled physical activity, regular outpatient visits according to the comprehensive care after myocardial infarction (CCMI) model (in Polish: “KOS-zawał”), administered by the National Health Fund of Poland. Additionally, before starting rehabilitation and immediately after completion, the patients performed an exercise stress test. Patients underwent daily cardiac rehabilitation for 4 weeks. The rehabilitation model (A, B, C or D) was selected according to the risk of cardiovascular events and physical capacity, following the guidelines of the European Society of Cardiology [15,16]. Patients were prescribed endurance training, resistance training, general exercise with frequency, session duration and intensity dependent on the rehabilitation model and individual health limitations [17].

After 8 weeks post-MI patients were contacted and scheduled for a follow-up visit consisting of physical examination, medical history update, blood sample collection for miRNAs analysis and follow-up echocardiographic examination. All investigations were completed under the same conditions for both the baseline and follow-up tests.

After the follow-up, the clinical and echocardiographic data of all initially enrolled 39 patients were evaluated. All 39 patients successfully completed the required cardiac rehabilitation program and the follow-up assessments. After follow-up visit patients were divided into two distinct groups based on echocardiographic criteria for reverse ventricular remodeling. All measurements were assessed independently by two experienced physicians:

- LV reverse remodeling (LVRR) Group (n = 10): patients showed reverse LV remodeling (defined as an absolute increase in LVEF ≥ 10% or a decrease in end-systolic LV volume of 15% or end-diastolic LV volume of 10% on TTE performed at the follow-up visit compared with the initial evaluation).
- Stable left ventricular status (SLVS**)** Group (n = 10): a clinical control, demonstrating a stable left ventricular status with minimal to no structural or functional echocardiographic changes compared to baseline. To ensure a clear clinical contrast with the LVRR group and account for standard echocardiographic inter-observer variability, SLVS was numerically defined as an absolute change in LVEF of <5% in either direction, combined with absolute changes in LV volumes (both ESV and EDV) of <10% compared to baseline.

After evaluation, 10 patients who met the criteria for LVRR and 10 patients who met the criteria for SLVS were matched by age and comorbidities and included in further miRNA analysis.

The remaining 19 patients from the initial cohort had intermediate echocardiographic trajectories or exhibited adverse LV remodeling and did not strictly meet the criteria for the LVRR or SLVS groups. Therefore, they were excluded from the molecular profiling phase. The detailed flow of patient enrollment, allocation, and analysis is presented in Fig 1. The final 20 patients (10 LVRR and 10 SLVS) were matched based on age, comorbidities, and standard medical therapy. However, exact gender matching was limited due to the availability of patients who met the strict group definitions.

**Fig 1:**
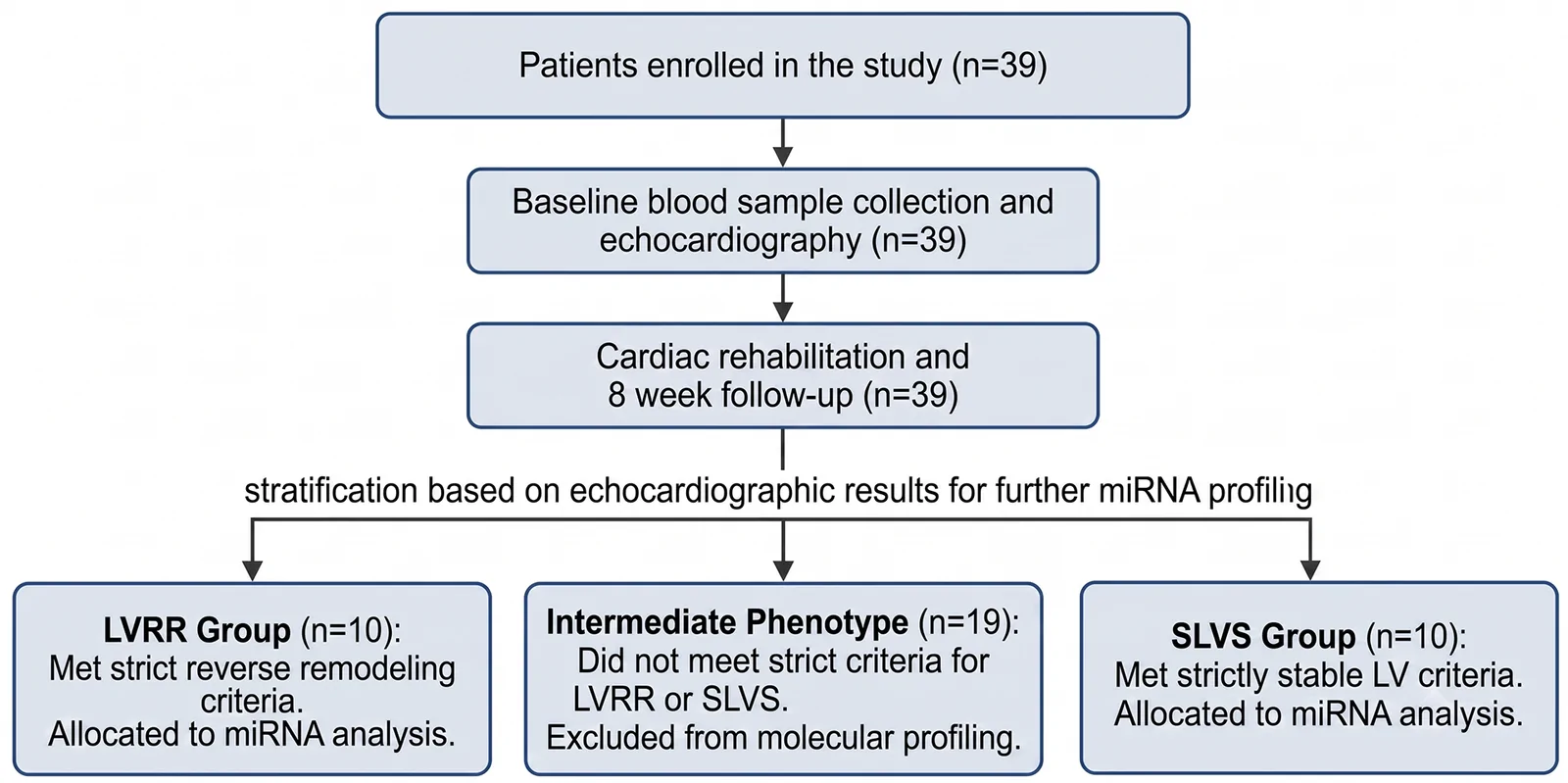
Study flow diagram and patient disposition. LVRR= left ventricle reverse remodeling; SLVS= stable left ventricle status.

### Data collection

Baseline demographics, medical history, clinical parameters, laboratory results were gathered from blood tests performed in the laboratory of our hospital, from medical records, from medical history collected from patients, and through physical examination at baseline and 8 weeks follow-up. All the data collected were blinded to the echocardiographic findings.

### Evaluation of ventricular remodeling

Echocardiographic examinations were performed by experienced echocardiography physician at day 3 and 8 weeks after the MI onset. Routine ultrasound and Doppler tissue images were obtained in the long and short axis views of the left ventricle, apical four-chamber view, two-chamber view, and long axis view using a Vivid Ultrasound echocardiography system manufactured by GE HealthCare. Conventional echocardiographic parameters were measured according to the recommendations of the American Society of Echocardiography/European Association of Cardiovascular Imaging (EACVI) guidelines [18,19].

Echocardiographic images were digitally recorded and analyzed using EchoPac software. All measurements were performed by two physicians independently, the mean value of each parameter was considered for further statistical analysis. LV volumes and LVEF were calculated by the biplane method of disks using 2-dimensional images and LV volumes were indexed to body surface area. Global longitudinal strain (GLS) was calculated using dedicated software, in the apical 4-chamber, 2-chamber, and long-axis views. Peak atrial longitudinal strain (PALS) was measured in the 4-chamber and 2-chamber apical views.

### Statistical analysis

Clinical data were analyzed using the PS IMAGO PRO 10 (IBM SPSS Statistics 29) software. Statistical significance was set at p < 0.05. To assess the distribution of quantitative variables, the Kolmogorov-Smirnov test was applied. For normally distributed data, mean and standard deviation were used to present the results, while median, 25 quartile and 75 quartiles (Q1; Q3) were reported for non-normally distributed data. To compare categorical values between two groups, Fisher-Exact Test or χ2 was used, while continuous variables were compared using T-student test or Mann-Whitney, as appropriate. To compare dependent variables, the paired samples T test or the Wilcoxon test was used, depending on the data distribution.

All miRNA profiling was conducted with the use of R custom scripts. For imputation the MSnbase R/Bioconductor package was used [20,21].

### Plasma samples and quality control

Peripheral blood samples were drawn at day 3 and 8 weeks after admission. Samples were immediately centrifuged at 5000 rpm for 10 minutes at 4°C. During the plasma isolation process, the supernatant was carefully aspirated by holding the pipette tip directly below the surface. The buffy coat was left intact to prevent contamination with leukocytes and platelets. After a visual inspection, all samples showing macroscopic signs of hemolysis were excluded. The resulting platelet-poor plasma was then divided into 500µl aliquots and frozen at −80°C until final assignment to groups (LVRR vs. SLVS) following an 8-week clinical evaluation. To prevent analytical batch effects, all selected portions were thawed and subjected to total RNA extraction and subsequent RT-qPCR profiling simultaneously within a single experimental batch. A total of 40 serum samples prospectively collected from 20 patients were analyzed.

### MiRNAs assay

Total RNA was extracted from 100 µL of plasma from 40 human samples using the MagMAX™ mirVana™ Total RNA Isolation Kit (Cat. No. A27828) according to the manufacturer’s instructions. To generate the pooled cohorts, equal volumes of plasma from each of the 10 patients per group were combined prior to RNA extraction. This ensured an equal representation of each individual in the final 4 pooled samples (R1 - 3 days after MI, LVRR group; R2 - 8 weeks after MI, LVRR group; C1 - 3 days after MI, SLVS group; C2 - 8 weeks after MI, SLVS group).

Total RNA was converted to cDNA and amplified using the TaqMan® Advanced miRNA cDNA Synthesis Kit (Cat. No. A28007). The cDNA was mixed with TaqMan™ Fast Advanced Master Mix and each reservoir of the TaqMan Advanced miRNA Human A Card (#A34714; Applied Biosystems) was loaded with 100 μL of prepared PCR reaction mix. The TaqMan Array Card is a 384-well microfluidic card that is prepared with dried-down TaqMan™ Assays/ with pre-formulated primer and probe sets for 380 human miRNAs, including an endogenous normalization control (miR-16-5p), as well as exogenous controls (cel-39-3p, ath-miR159a). MiR-16 transcript was used as a normalization signal.

### MiRNA Profiling

For miRNA profiling in analyzed samples raw threshold cycle (Ct) values generated by Applied Biosystem ViiA™ 7 Software were used. To obtain absolute quantities of all assayed miRNAs the list was filtered to remove all undetected or unreliable (with Ct > 35) miRNAs. Data normalization was executed using Global Mean Normalization, wherein the sample-specific mean Ct values of all fully quantified targets (Ct < 35) were subtracted from individual target Ct values to yield normalized expression levels (ΔCt = Ct _mean_ - Ct_target_). The rationale of this analysis is that a normalization factor based on geomean uses a factor that indicates central tendency of a sample values and thus should remove any nonspecific variation between samples. For convenient interpretation of miRNA levels geomean normalized Ct values were transformed by subtracting them from constant size factor that was determined as rounded max Ct value of whole dataset plus 1. Thus, the norm. Ct does not range in negative values and is proportional to log2 of miRNA copies when assuming 100% efficiency of PCR reaction. Relative fold changes between experimental pools were calculated using the 2^- ΔΔCt^ method. As of primary screening characteristics of our analysis, we removed only miRNAs with unreliable and undetectable levels in sample R1. Missing values in high-throughput RT-qPCR profiling typically represent non-random, left-censored data, where microRNA abundance falls below the assay’s Limit of Detection (LOD), rather than random technical failures. To prevent the severe selection bias caused by complete-case analysis, missing values were imputed using the probabilistic minimum imputation (minProb) algorithm. The minProb method models missing values by drawing from a Gaussian distribution centered on the minimal quantified intensity across the dataset, scaled by a shrinkage factor effectively assigning a realistic upper-bound value corresponding to the background detection limit. This approach preserves the biological gradient of low-abundance miRNAs while preventing false inflation of fold-change calculations. All miRNA profiling was conducted with the use of R custom scripts.

### MiRNA Biomarker Candidate Selection

The normalized Ct values reflecting the log2 miRNA abundance in plasma were used to compare the accumulation levels of individual miRNAs between LVRR group and the control group at different time points. The differences between compared patients’ groups were performed as in ΔΔCt method, thus the log_2_ fold change (FC) was calculated by subtracting the normalized Ct value of control group from normalized Ct value of rehabilitation responding patients’ group (Ct_R1 –_ Ct_C1_). miRNA candidate biomarkers were selected mainly based on FC criterion. Because screening was performed on single pooled samples per group, differential expression could not be evaluated via inferential statistics. Instead, candidate selection was guided by global Pearson correlation profiling followed by a tiered filtering framework anchored to physical cycle threshold shifts (ΔCt). All plot representations of biomarker candidates were performed with the use of R package ggplot2.

### Gene ontology and pathway analysis of miRNA target genes

Lists of predicted miRNA target genes were extracted from miRTarBase (v 10.0) (http://mirtarbase.cuhk.edu.cn/~miRTarBase/miRTarBase_2025). The pathway overrepresentation analysis was performed with KEGG pathways annotations using the enrichKEGG function in R/Bioconductor package clusterProfiler [22]. Top 10 significant pathways were selected, and network analysis of gene union between enriched pathways was performed. The network representation was prepared with the use of custom R scripts implementing R packages: igraph [23] and ggraph [24].

### MiRNA target genes network generation

For miRNA target genes, a network generation list of genes interacting with more than 3 miRNAs with high potential of being a candidate biomarker was selected. The network representation was prepared with the use of custom R scripts implementing R packages: igraph [23] and ggraph [24].

## Results

### Clinical data

A total of 20 patients (mean age 57 ± 9 years) were enrolled in this study, 10 in each group. Table 2 presents the patients’ characteristics at baseline and 8 weeks follow up.

**Table 2:** Characteristics of study population at baseline and 8 weeks after MI. For qualitative variables, data were presented as counts and percentages, for quantitative variables as mean (SD) or median (Q1; Q3), depending on the distribution of the data. ASA – acetylsalicylic acid; ACEI - angiotensin-converting-enzyme inhibitor; BMI – body mass index; cTnI – cardiac troponin I; HbA1c - glycated hemoglobin; HR – heart rate; HDL – high density lipoprotein; IFG – impaired fasting glucose; IRA – infarct-related artery; LAD – left anterior descending artery; LCx - left circumflex artery; LDL – low density lipoprotein; NT-proBNP - N-terminal prohormone of brain natriuretic peptide; P2Y12 - P2Y12 inhibitor; RCA – right coronary artery; SGLT2i - sodium-glucose transport protein 2 inhibitor; SBP – systolic blood pressure.

Total n = 20			
Variable		Baseline	8 weeks
**Age,** years		57 ± 9	
**Sex,** male gender, **% (n)**		70 (14)	
**BMI,** kg/m2		29.1 ± 4.2	28.6 ± 4.4
**SBP,** mmHg		125 ± 14	124 ± 13
**HR,** bpm		73 ± 9	66 ± 9
**Physical capacity,** METs		6.8 ± 1.9	7.7 ± 2.4
**Past medical history**			
**Nicotine use, % (n)**		45 (9)	5 (1)
**Hypertension, % (n)**		50 (10)	
**Dyslipidemia, % (n)**		90 (18)	
**Glucose metabolism disorders, % (n)**		55 (11)	
	IFG	25 (5)	
	diabetes	30 (6)	
**Myocardial infarction**			
**STEMI/ NSTEMI**		70 (14)/ 30 (6)	
Ischemia reperfusion time*, % (n)			
	<2 hours	30 (6)	
	2-24 hours	45 (9)	
	>24 hours	20 (4)	
**IRA, % (n)**			
	LAD	55 (11)	
	LCx	15 (3)	
	RCA	30 (6)	
**Blood analysis**			
**Fasting glucose, mg/dl**		112 (96; 151)	105 (93; 125)
**HbA1c, %**		5.5 (5.3; 6.6)	
**Total cholesterol, mg/dl**		212 ± 52	134 ± 32
**LDL, mg/dl**		148 ± 51	72 ± 26
**HDL, mg/dl**		41 (39; 46)	42 (40; 47)
**Triglicerydes, mg/dl**		126 ± 56	100 ± 41
**NT-proBNP pg/ml**		608 (188; 1461)	
**cTnI 1** ^**st**^ **, ng/l**		97 (41; 387)	
**cTnI 2** ^**nd**^ **, ng/l**		12902 (3017; 43864)	
**Medication**			
**ASA**		100 (20)	95 (19)
**P2Y12 inhibitor**		100 (20)	100 (20)
**ACE-I**		85 (17)	85 (17)
**Beta-blockers**		70 (14)	70 (14)
**Aldosterone antagonist**		15 (3)	15 (3)
**Statins**		100 (20)	100 (20)
**SGLT2i**		45 (9)	45 (9)

The majority of the study population were male (70%). Among the subjects, 50% had hypertension, while 55% had impaired glucose metabolism (IFG or DM2). Dyslipidemia was present in 90% of patients. Additionally, only 10% of patients had normal weight based on a body mass index (BMI) up to 24.99 kg/m2, the rest of the population was overweight (BMI 25–29.99 kg/m2) or obese (BMI > 30 kg/m2), 50% and 40% of patients, respectively. A history of active or past nicotine use was noted in 9 patients, representing 45% of the study participants. The most prevalent infarct-related artery was LAD, accounting for 55% of the AMIs. The remaining vessels treated with PCI were RCA and LCx, in 30% and 15% of the cases, respectively.

The comparison of patients in the LVRR and SLVS groups is presented in Table 3. Patients in the two groups did not statistically differ in age, sex, past medical history or laboratory results. All patients were treated with an optimal pharmacological therapy for coronary heart disease. There were no changes in pharmacotherapy over the 8-week follow-up, except for necessary dose up-titrations and 1 patient, who had ASA intolerance. The entire population was prescribed a statin. The majority of patients were treated with an ACE inhibitor (90% in the LVRR group and 80% in the SLVS group, p = 0.53), and a beta-blocker (80% in the LVRR group and 60% in the SLVS group, p = 0.33). An aldosterone antagonist was used in only three subjects in the LVRR group compared to none in the SLVS group (p = 0.06). In addition, more patients in the LVRR group were treated with an SGLT2 inhibitor (70% vs. 20%); however, none of these differences reached statistical significance (p = 0.07).

**Table 3:** Comparison of demographics and clinical characteristics parameters between LVRR and SLVS group. For qualitative variables, data were presented as counts and percentages, for quantitative variables as mean (SD) or median (Q1; Q3), depending on the distribution of the data. ASA – Acetylsalicylic acid; ACEI - angiotensin-converting-enzyme inhibitor; BMI – body mass index; cTnI – cardiac troponin I; HbA1c - glycated hemoglobin; HR – heart rate; HDL – high density lipoprotein; IFG – impaired fasting glucose; IRA – infarct-related artery; LAD – left anterior descending artery; LCx - left circumflex artery; LDL – low density lipoprotein; NT-proBNP - N-terminal prohormone of brain natriuretic peptide; RCA – right coronary artery; SGLT2i - sodium-glucose transport protein 2 inhibitor; SBP – systolic blood pressure; *Comparison between two groups used Fisher-Exact Test or Chi-square for categorical data and T-student test or Mann-Whitney test for continuous variables. First p value refers to baseline comparison between LVRR and SLVS Groups at baseline, second value refers to comparison between LVRR and SLVS after 8 weeks post-MI.

		LVRR n = 10		SLVS n = 10		
		Baseline	8 weeks	Baseline	8 weeks	P value*
**Age,** years		57 ± 10		58 ± 8		0.83
**Sex,** male gender, **% (n)**		50 (5)		90 (9)		0.14
**BMI,** kg/m2		30.5 ± 4	29.5 ± 4.3	27.6 ± 4	27.8 ± 4.6	0.12; 0.40
**SBP,** mmHg		121 ± 13	122 ± 9	130 ± 14	126 ± 17	0.13; 0.58
**HR,** bpm		75 ± 12	63 ± 10	71 ± 6	68 ± 9	0.44; 0.29
**Physical capacity,** METs		6.6 ± 2.1	7.5 ± 2.4	7.2 ± 1.9	8.2 ± 2.5	0.51; 0.55
**Past medical history**						
**Nicotine use, % (n)**		30 (3)	0 (0)	60 (6)	10 (1)	0.14; 1.00
**Hypertension, % (n)**		50 (5)		50 (5)		1.00
**Dyslipidemia, % (n)**		90 (9)		90 (9)		1.00
**Glucose metabolism disorders, % (n)**		70 (7)		40 (4)		0.15
	IFG	20 (2)		30 (3)		
	diabetes	50 (5)		10 (1)		
**Myocardial infarction**						
**STEMI/ NSTEMI**		80 (8)/ 20 (2)		60 (6)/ 40 (4)		0.63
Ischemia reperfusion time*, % (n)						0.97
	<2 hours	30 (3)		30 (3)		
	2-24 hours	40 (4)		50 (5)		
	>24 hours	20 (2)		20 (2)		
**IRA, % (n)**						0.58
	LAD	60 (6)		50 (5)		
	LCx	20 (2)		10 (1)		
	RCA	20 (2)		40 (4)		
**Blood analysis**						
**Glucose, mg/dl**		126 (105; 184)	112 (103; 141)	99 (88; 123)	102 (91; 111)	0.97; 0.15
**HbA1c, %**		5.3 (5.25; 7.90)		5.5 (5.35; 5.93)		0.95
**Total cholesterol, mg/dl**		213.30 ± 50.51	128 ± 23	211 ± 57	140 ± 38	0.91; 0.47
**LDL, mg/dl**		144 ± 51	65 ± 18	152 ± 54	78 ± 32	0.72; 0.30
**HDL, mg/dl**		41 (40; 43)	42 (39; 54)	40 (35; 47)	42 (39; 45)	0.51; 0.73
**Triglicerydes, mg/dl**		147 ± 64	101 ± 41	103 ± 36	100 ± 43	0.085; 0.996
**NT-proBNP**		934 (349; 2272)		372 (95; 867)		0.11
**cTnI 1st, median (Q1; Q3)**		97 (81; 918)		87 (6; 681)		0.39
**cTnI 2nd, median (Q1; Q3)**		10127 (3181; 50587)		13870 (2070; 35991)		0.57
**Medication**						
**ASA**		100 (10)	100 (10)	100 (10)	100 (10)	
**P2Y12 inhibitor**		100 (10)	100 (10)	100 (10)	100 (10)	
**ACE-I**		90 (9)	90 (9)	80 (8)	80 (8)	0.53
**Beta-blockers**		80 (8)	80 (8)	60 (6)	60 (6)	0.33
**Aldosterone antagonist**		30 (3)	30 (3)	0 (0)	0 (0)	0.06
**Statins**		100 (10)	100 (10)	100 (10)	100 (10)	
**SGLT2i**		70 (7)	70 (7)	20 (2)	20 (2)	0.07

### Echocardiography results

The mean LVEF at baseline was measured at 48.35 ± 8.18%. The value of LVEF and GLS measured at baseline was significantly different between the groups (p = 0.013 and p < 0.001, respectively). Other parameters of LV function, such as LVESVi, LVEDVi, LVEDDi did not differ between the groups at baseline (Table 4).

**Table 4:** Echocardiographic parameters in studied groups at baseline and after 8 weeks. Quantitative data presented as mean ± SD and qualitative data presented as % (n). LVEDVi - indexed left ventricle end diastolic volume; LVEDDi - indexed left ventricle end-diastolic diameter; LVEF – left ventricular ejection fraction; LVESVi - indexed left ventricle end-systolic volume; GLS – global longitudinal strain; PALS – peak atrial longitudinal strain *Difference between LVRR and SLVS Groups.

	Total n = 20	LVRR n = 10	SLVS n = 10	P Value*
**Baseline**				
**LVEF, %**	48 ± 8	44 ± 7	53 ± 7	0.013
**LVESVi, ml/m2**	28.9 ± 7.2	31.4 ± 8.2	26.5 ± 5.5	0.139
**LVEDVi, ml/m2**	58.1 ± 9.1	57.5 ± 9.8	58.8 ± 8.8	0.76
**PALS**	27 ± 7	25 ± 5	29 ± 7	0.12
**GLS**	−14.4 ± 3.5	−12.1 ± 2.5	−16.8 ± 2.7	<0.001
**LVEDDi**	2.48 ± 0.25	2.43 ± 0.29	2.57 ± 0.22	0.39
**Regional contractility impairment, % (n)**	75 (15)	100 (10)	50 (5)	0.033
**8 weeks**				
**LVEF, %**	54 ± 7	55 ± 6	54 ± 7	0.69
**LVESVi, ml/m2**	25.6 ± 6.8	25.4 ± 8.1	25.8 ± 5.8	0.90
**LVEDVi, ml/m2**	54.6 ± 11.7	53.9 ± 13	55.4 ± 10.8	0.78
**PALS**	30 ± 7	30 ± 7	30 ± 7	0.99
**GLS**	−16.5 ± 2.4	−16.2 ± 2.6	−16.7 ± 2.3	0.62
**LVEDDi**	2.49 ± 0.24	2.51 ± 0.27	2.48 ± 0.21	0.78
**Regional contractility impairment, % (n)**	40 (8)	30 (3)	50 (5)	0.65

The SLVS group had a significantly higher EF at study inclusion and had significantly better GLS (EF 53% vs 44% and GLS −16.8 vs −12.1, respectively). Regional contractility impairment at baseline was observed in 100% of LVRR patients and was reversed in 70% of them after 8 weeks. No change in the incidence of left ventricular regional contractility dysfunction was observed in the SLVS group and was equal to 50%. Consistent with the study design, echocardiographic parameters at baseline and 8 weeks did not differ significantly in the SLVS group, while in the LVRR group we observed a significant improvement in EF, GLS, PALS and a reduction in LVESVi and LVEDDi. The only parameter that was not significantly different in the reverse remodeling group was LVEDVi (p = 0.038) (Table 5).

**Table 5:** Echocardiographic parameters at baseline and after 8 weeks post MI. Quantitative data presented as mean ± SD.

LVRR n = 10				SLVS n = 10		
	Baseline	8 weeks	p value*	Baseline	8 weeks	p value*
**LVEF, %**	44.00 ± 6.72	55.00 ± 6.13	<0.001	52.7 ± 7.35	53.80 ± 7.13	0.10
**LVESVi, ml/m2**	31.4 ± 8.2	25.4 ± 8.1	<0.001	26.5 ± 5.5	25.8 ± 5.8	0.42
**LVEDVi, ml/m2**	57.5 ± 9.8	53.9 ± 13	0.125	58.8 ± 8.9	55.4 ± 10.8	0.038
**LVEDDi, cm/m2**	2.43 ± 0.29	2.51 ± 0.27	0.032	2.57 ± 0.22	2.48 ± 0.21	0.16
**PALS**	24.55 ± 5.21	30.00 ± 7.28	0.010	29.10 ± 7.07	29.95 ± 6.59	0.49
**GLS**	−12.05 ± 2.50	−16.16 ± 2.64	<0.001	−16.77 ± 2.65	−16.73 ± 2.33	0.92

LVEDDi - indexed left ventricle end-diastolic diameter; LVEDVi - indexed left ventricle end diastolic volume; LVEF – left ventricular ejection fraction; LVESVi - indexed left ventricle end-systolic volume; GLS – global longitudinal strain; PALS – peak atrial longitudinal strain

*Comparison of means between paired continuous variables at baseline and at 8 weeks using paired samples T- test.

### MiRNA analysis

To identify miRNAs differentially accumulated in the serum of the two patient groups, we first evaluated the accumulation levels in each of the four pooled sera samples. Notably, several miRNAs included in the assay were not detected across all samples. Of the 375 miRNAs represented on the plate, 107 were not quantified in any of the samples, and others were detected in only one, two or three samples (S1 Table). Analysis of miRNA detection revealed that a greater number of miRNAs were identified at both time points in the patients who exhibited reverse remodeling, compared with the SLVS group which served as the control group (Fig 2 and S1 Table). In summary, 157 miRNAs were quantified in all four samples. Sample R1 showed the highest number of uniquely detected miRNAs (23), whereas additional 10 miRNAs were quantified exclusively in samples obtained from patients from LVRR group (R1 and R2). To narrow the analysis to candidate miRNAs with a biomarker potential we excluded all miRNAs that were not detected or unreliable (had C_t_ ≥ 35) in the baseline sample of the LVRR (R1).

**Fig 2:**
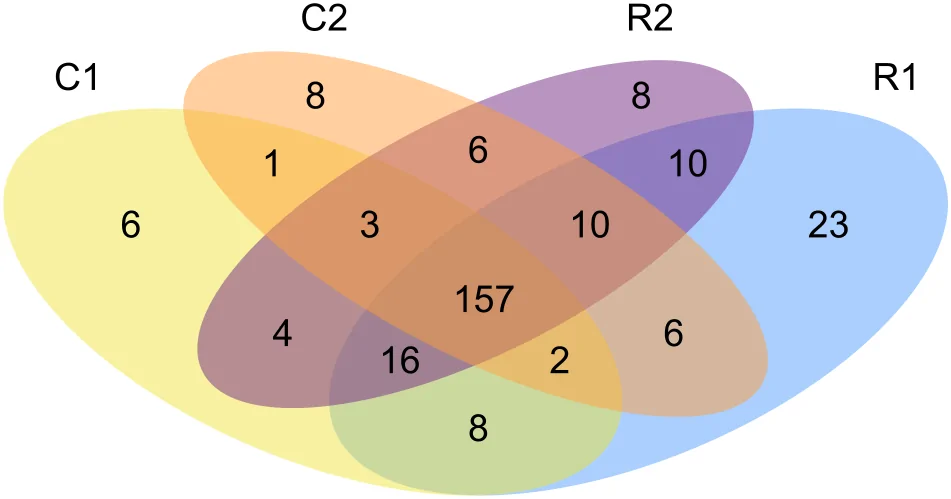
Distribution of Shared and Unique Circulating miRNAs.

The Venn diagram illustrates the number of shared and unique miRNA molecules detected across study groups and time points. C1 - SLVS group at baseline; C2 - SLVS group at 8 weeks; R1 - LVRR group at baseline; R2 - LVRR group at 8 weeks.

This approach ensured that our analysis focused on miRNAs that could be reliably quantified in the targeted group of patients. To avoid excluding potential biomarkers with elevated accumulation levels in the LVRR group on day 3, and to enable efficient comparison between the patient’s groups, values were imputed for all miRNAs that were not quantified in the assay.

After initial filtering, normalization and imputation of the data we compared the miRNA accumulation profiles between distinct patients’ groups (Figs 2 and 3). Correlation analysis revealed clear differences in miRNA accumulation between patients who experienced reverse remodeling and patients in the control group. The strongest correlation was observed between miRNA accumulation profiles for control group samples at two time points (Pearson’s Correlation r = 0.80), whereas the correlations between LVRR patients and control group patients at both time points (after 3 days and after 8 weeks) was markedly lower (r = 0.67 and r = 0.75 respectively).

**Fig 3:**
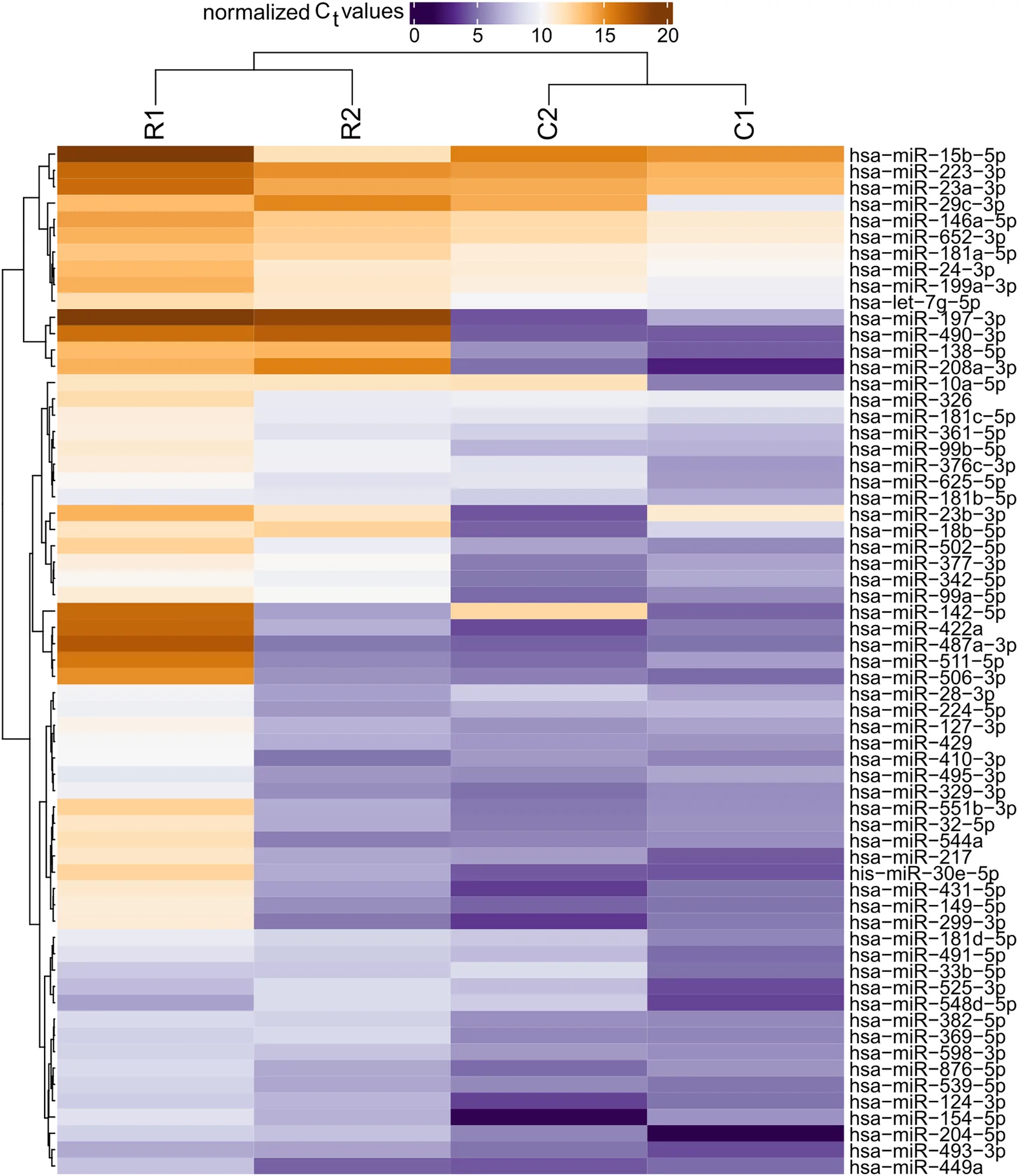
Heatmap of associations representing miRNAs expression. The heat map shows the associations and expression levels of miRNAs. A correlation analysis was performed on normalized Ct values to assess overall differences between the LVRR and SLVS groups at two time points. R1 - LVRR group at baseline; R2 - LVRR group at 8 weeks; C1 - SLVS group at baseline; C2 - SLVS group at 8 weeks.

Considering that the initial miRNA accumulation profiles showed predominant miRNAs up-regulation- and that cardiac infarction is known to induce the release of miRNAs into the blood system – we focused our analysis on miRNAs with elevated levels when compared to the SLVS group. Hence, a miRNA was considered a candidate biomarker if its level on day 3 after MI was considerably higher in patients who later demonstrated LVRR (Figs 3 and 4). Using these criteria, more than 35 miRNAs were identified as potential biomarker candidates. To refine the list for further validation and exploratory analyses, we applied additional criteria based on assay reliability. To guarantee that imputed baseline values did not artifactually drive candidate selection, one of the criteria for miRNAs stratification was based on quantification completeness. MiRNAs were categorized according to their potential as biomarkers as follows: (i) high potential – the fold change of miRNA is higher or equal 8 (corresponding to a difference of 3 C_t_ between groups), with a minimal normalized C_t_ of 10 in the R1 sample. Additionally, C_t_ values were not imputed in the baseline samples (R1 and C1), ensuring robust initial quantification, (ii) mid potential – the fold change of miRNA is higher or equal 5 (corresponding to a difference of 2.32 C_t_ between groups) with a minimal normalized C_t_ of 5 in the R1 sample. Additionally, C_t_ values were not imputed in C1 sample, (iii) low potential – the fold change of miRNA is higher or equal 5 (corresponding to a difference of 2.32 C_t_ between groups) and normalized C_t_ is below 5 for R1sample.

**Fig 4:**
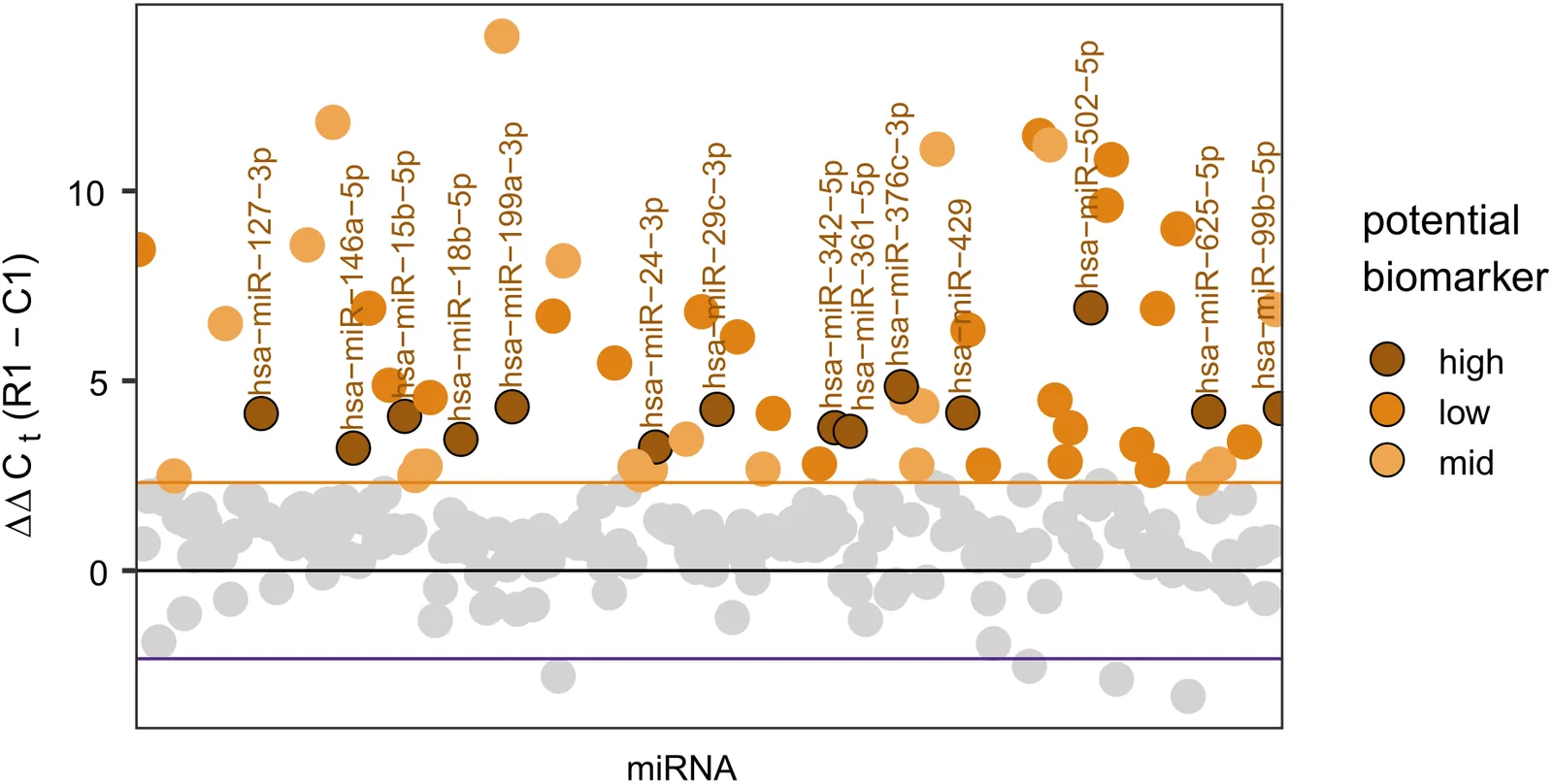
Categorization of miRNAs by Biomarker Potential. Graph showing miRNA expression by biomarker potential according to established criteria. High biomarker potential = the FC of miRNA is higher or equal 8 (difference of 3 Ct between groups) and the minimal normalized Ct equals 10 for the LVRR group of patients measured at baseline. The ΔΔCt method is used to quantitatively determine the relative abundance of miRNA by comparing the LVRR (R1) and SLVS (C1) groups. Ct – cycle threshold; FC – fold change; LVRR – left ventricular reverse remodeling; MI – myocardial infarction; SLVS – stable left ventricle status.

Consequently, we selected 14 miRNAs that met the inclusion criteria and demonstrated high potential of being candidate biomarkers of LVRR after MI: miR-15b-5p, miR-24-3p, miR-29c-3p, miR-99b-5p, miR-127-3p, miR-146a-5p, miR-199a-3p, miR-361-5p, miR-502-5p, miR-625-5p, miR-376c-3p, miR-18b-5p, miR-342-5p, and miR-429 (Fig 4 and Table 6).

**Table 6:** Expression profiles and relative fold changes of 14 high-potential candidate miRNAs in patients with left ventricular reverse remodeling (LVRR) and stable left ventricular status (SLVS).

miRNA	Normalized expression level (transformed Ct)							
	SLVS day 3 (C1)	SLVS week 8 (C2)	LVRR day 3 (R1)	LVRR week 8 (R2)	Log2 FC (LVRR vs SLVS at Day 3)	Log2 FC (LVRR vs SLVS at Week 8)	Log2 FC (SLVS Week 8 vs Day 3)	Log2 FC (LVRR Week 8 vs Day 3)
hsa-miR-15b-5p	14.88	15.45	18.94	11.81	4.07	−3.64	0.57	−7.14
hsa-miR-18b-5p	8.16	**5.13**	11.62	12.46	3.46	7.33	−3.02	0.85
hsa-miR-24-3p	10.25	11	13.51	11.29	3.25	0.29	0.75	−2.22
hsa-miR-29c-3p	9.23	14.08	13.47	15.24	4.25	1.16	4.85	1.76
hsa-miR-99b-5p	6.87	6.96	11.14	9.62	4.27	2.66	0.09	−1.52
hsa-miR-127-3p	6.35	5.83	10.49	6.85	4.14	1.02	−0.52	−3.64
hsa-miR-146a-5p	11.19	12.08	14.42	12.77	3.23	0.7	0.89	−1.64
hsa-miR-199a-3p	9.59	10.8	13.9	11.43	4.32	0.63	1.21	−2.47
hsa-miR-342-5p	6.53	**4.49**	10.3	9.61	3.77	5.12	−2.05	−0.69
hsa-miR-361-5p	7.15	8	10.81	8.86	3.67	0.86	0.86	−1.96
hsa-miR-429	5.87	**2.89**	10.03	**6.05**	4.16	3.16	−2.98	−3.98
hsa-miR-502-5p	5.58	6.36	12.5	9.51	6.92	3.16	0.78	−2.99
hsa-miR-625-5p	6.09	8.97	10.28	8.8	4.19	−0.17	2.88	−1.48
hsa-miR-376c-3p	6.02	8.87	10.87	9.67	4.85	0.79	2.85	−1.2

Values in columns C1, C2, R1, and R2 represent transformed normalized cycle threshold (Ct) values, which are proportional to the log2 of miRNA copies. Values marked in bold denote imputed data. FC – fold change; LVRR – left ventricular reverse remodeling; SLVS – stable left ventricular status.

In the next step, we evaluated the lists of genes predicted to interact with the miRNAs of high biomarker potential. An in-depth analysis of miRNA: target interactions revealed several genes that function as hub genes interacting with many miRNAs from the candidate biomarker set (Fig 5). Notably, the genes with the highest number of interactions were two transcription regulators (Zinc Finger ZZ-Type Containing 3 *[ZZZ3*] and Specificity Protein 1 [*SP1*]).

**Fig 5:**
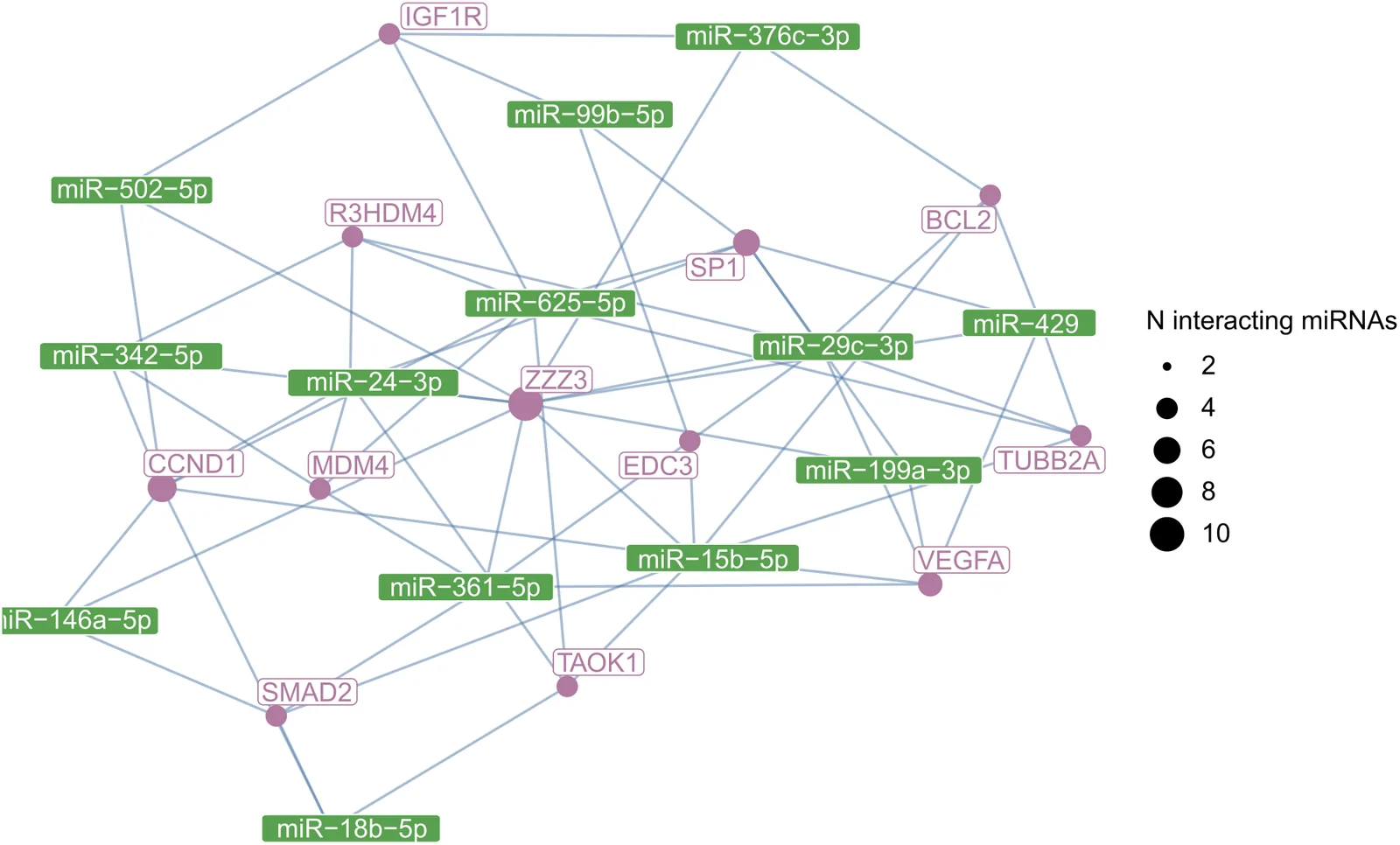
Network analysis of miRNA:target gene interactions.

For miRNA target genes, a network generation list of genes interacting with more than 3 miRNAs with high potential of being a candidate biomarker was selected. The network visualizes the interactions between 13 miRNA biomarkers with high potential and their predicted target genes. The results identify the ZZZ3 and SP1 genes as central “hubs” interacting with the largest number of the miRNAs studied.

BCL2 – B cell lymphoma 2; CCND1 – Cyclin D1; EDC3 – Enhancer Of mRNA Decapping 3 Homolog; IGF1R - Insulin Like Growth Factor 1 Receptor; MDM4 – Murine double minute 4; N – number of miRNAs interacting with the hub gene; R3HDM4 - R3H Domain Containing 4; SMAD2 – SMAD Family Member 2; SP1 – specificity protein 1; TAOK1 – Thousand And One Kinase 1 (Serine/Threonine-Protein Kinase TAO1); TUBB2A - Tubulin Beta 2A Class IIa; VEGFA – Vascular Endothelial Growth Factor A; ZZZ3 – Zinc Finger ZZ-Type Containing 3;

In the next step of the exploratory analysis, we performed Gene Ontology (GO) enrichment analysis on the predicted target genes of the identified candidate miRNA biomarkers. This investigation revealed that the top enriched biological processes were associated with several signaling pathways like *MAPK, PI3K-AKT, FOXO, p53, AGE-RAGE* signaling pathways (Fig 6).

**Fig 6:**
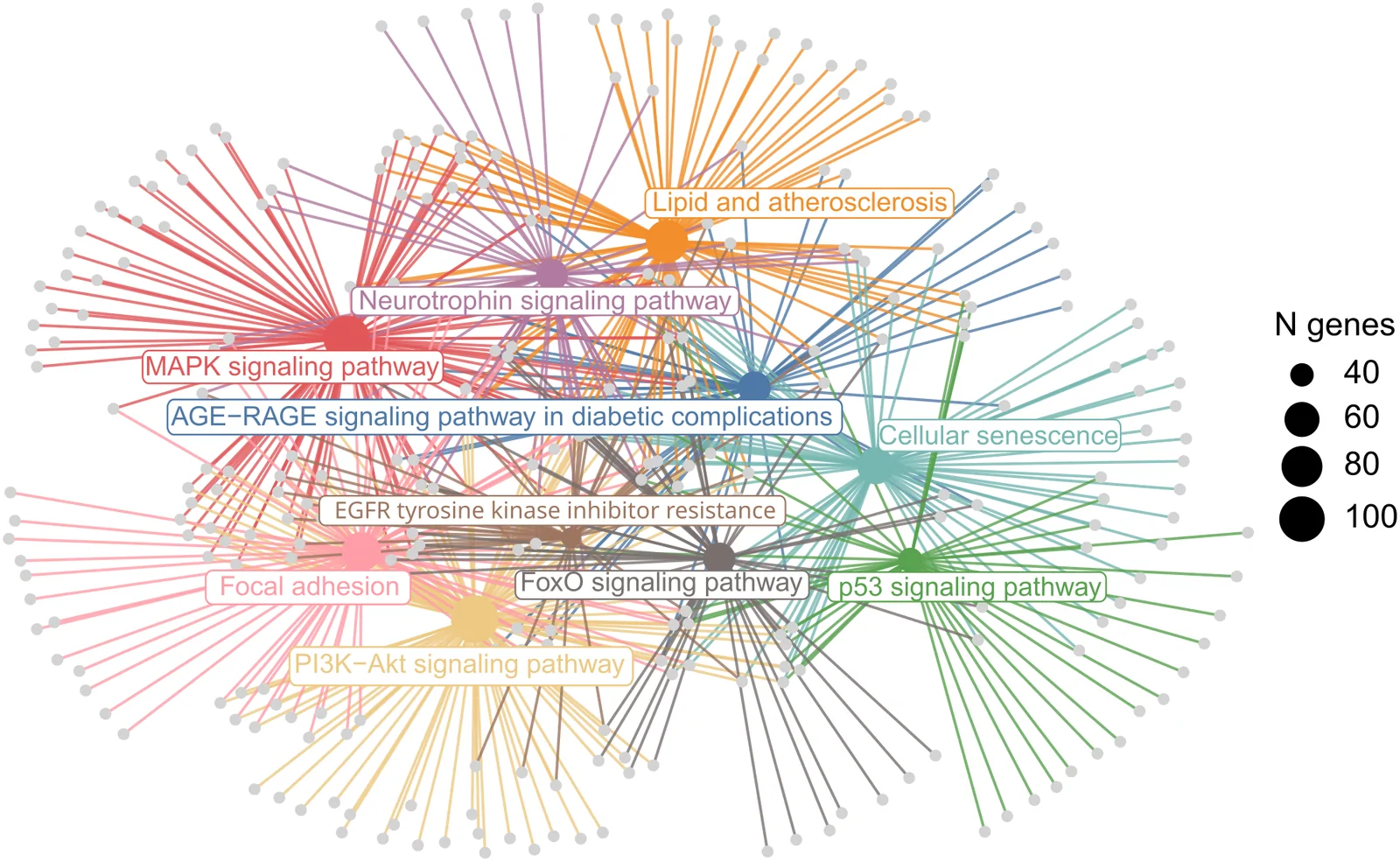
Functional Enrichment Analysis of Candidate miRNA Target Genes.

The figure illustrates the main biological processes related to genes interacting with potential biomarker miRNAs. A Gene Ontology (GO) enrichment analysis was performed to functionally map the mechanisms of cardiac reverse remodeling. The results indicate that these genes are primarily involved in the regulation of fatty acid metabolism and cellular stress responses.

## Discussion

The purpose of this study was to gain a deeper understanding of the role of miRNAs in the process of reverse cardiac remodeling after myocardial infarction. Therefore, we evaluated the peripheral blood miRNA profile in patients who demonstrated improvement in LV structure and function during follow-up compared with those who showed no change in myocardial structure and function according to our predefined remodeling criteria. Our aim was to identify candidate miRNAs that could potentially distinguish patients with a more favorable prognosis after MI in terms of LV muscle reverse remodeling. In our analysis, we identified several miRNAs with higher baseline accumulation based on descriptive thresholds in patients who subsequently exhibited reverse remodeling.

To characterize the prognostic value of baseline miRNA expression, we examined a comprehensive panel of 375 circulating miRNAs rather than restricting our analysis to candidates previously reported in the literature. Plasma miRNA levels were quantified using RT-qPCR, a reliable and reproducible method for assessing circulating miRNAs [25]. A critical consideration in circulating miRNA profiling of pooled samples is the handling of unquantified signals. Simply discarding unquantified miRNAs risks excluding highly specific candidate biomarkers that are below LOD in control cohorts (C1 and C2) but robustly induced under pathophysiological conditions or during left ventricular reverse remodeling (R1 and R2). By utilizing minProb imputation, we maintained the capacity to detect this accumulation shift.

Considering that the initial miRNA accumulation profiles were dominated by up-regulated miRNAs – and that cardiac infarction is known to induce the release of miRNAs into the bloodstream we decided to study miRNAs with elevated levels when compared to the SLVS patients’ group. Accordingly, a miRNA was considered a potential biomarker if its level on day 3 post-MI was considerably higher in the patients who later showed a positive response to the rehabilitation and optimal medical treatment (Figs 3 and 4).

Using these criteria, we identified 14 candidate miRNAs with high biomarker potential: miR-15b-5p, miR-24-3p, miR-29c-3p, miR-99b-5p, miR-127-3p, miR-146a-5p, miR-199a-3p, miR-361-5p, miR-502-5p, miR-625-5p, miR-376c-3p, miR-18b-5p, miR-342-5p, and miR-429.

### Temporal dynamics of miRNA expression

The LVRR cohort was characterized by a significantly lower baseline LVEF and GLS compared with the SLVS group, indicating greater initial myocardial damage. However, the temporal dynamics of our 14 candidate miRNAs may counter the hypothesis of passive molecular leakage from necrotic tissue. If these elevated baseline levels simply reflected a larger initial infarct, their circulating signal would be expected to undergo a rapid, uniform downregulation across all patients as the acute injury resolves. Instead, we observed three distinct biological trajectories suggesting an active, coordinated epigenetic program driving post-MI recovery:

1. **Sustained regulation (11 out of 14 miRNAs)**

Most candidates (11 of 14) met the criteria for high biomarker potential without requiring data imputation at any time point. In the LVRR group, they exhibited a robust peak at day 3, followed by sustained, quantifiable expression at 8 weeks. This suggests that these miRNAs are integral to ongoing structural rescue and scar maturation, rather than being transient footprints of acute ischemia.

**2. Asymmetrical downregulation in SLVS group (miR-18b-5p and miR-342-5p)**

Only 3 of the 14 candidates required any data imputation. For miR-18b-5p and miR-342-5p, imputation was necessary exclusively in the SLVS group at the 8-week follow-up (C2), while their expression remained robustly detectable in the LVRR group. This asymmetrical silencing in non-responders contradicts the theory of a universal, necrosis-driven temporal changes in miRNAs expression. We hypothesize that specific pro-survival pathways are actively maintained during reverse remodeling but extinguish in patients exhibiting stable left ventricular morphology.

**3. The acute “on/off” switch (miR-429)**

Uniquely, miR-429 required imputation at two time points. It was sharply upregulated exclusively during the acute phase in the LVRR group (Day 3) but fell below detection limits by week 8 in both cohorts. This dynamic aligns with its role as an acute hypoxia regulator, reflecting a “critical window” where early anti-apoptotic signals are initiated under stress and physiologically shut down once oxygen homeostasis is restored.

This dynamic suggests that cardiac reverse remodeling requires an acute epigenetic response – a ‘critical window’ during the early post-infarction phase – to successfully activate pro-survival, pro-angiogenic, and anti-apoptotic pathways (such as PI3K-Akt and FoxO). Time-dependent nature of miRNA expression post-MI observed in our clinical cohort was mentioned in recent preclinical in vivo models. In a rat model of permanent coronary occlusion, miRNA signatures underwent rapid temporal shifts that strictly correlated with the histological progression of inflammation and fibrotic remodeling [26]. During the early acute phase (24–72 h), cell death and hypoxia drove significant miRNA modulations and robust fibrotic gene upregulation. Crucially, a subsequent partial return of certain miRNAs to baseline levels by 1 month was observed, mirroring our clinical data and confirming that these molecular responses are strictly tied to active phases of tissue repair rather than passive necrotic clearance.

### Functional roles of candidate miRNAs: insights from existing literature

To gain further insight into their functional role in myocardial remodeling process, we reviewed existing literature on the candidate miRNAs. Most of the identified miRNAs have previously been implicated in the pathways relevant to post-MI cardiac remodeling. Based on the literature reports, these miRNAs can be broadly categorized into four groups: 1) upregulated in post-MI reverse remodeling processes, 2) downregulated in post-MI reverse remodeling processes, 3) results are conflicting depending on the experiment and context, and 4) no reports to date in post-MI remodeling, association with other cardiovascular diseases.

**1. Positive Regulators (Upregulated in Regenerative Processes)**

miRNAs such as **miR-99b-5p**, **miR-199a-3p**, **miR-342-5p**, and **miR-625-5p** exhibit cardioprotective roles in post-MI remodeling. These miRNAs have the potential to be attractive therapeutic targets.

In a study by Kane et al., it was shown that **miR-99b** is one of the miRNAs that can potentiate the differentiation of endothelial cells (ECs) from pluripotent human embryonic stem cells (hESCs), enhancing therefore angiogenesis. Moreover, the same study demonstrated that after transplantation of pluripotent ECs overexpressing miR-99b into the muscle of immunodeficient mice subjected to limb ischemia, neovascularization and blood flow recovery were promoted [27]. **MiR-99b-5p** was one of downregulated miRNAs in cardiac extracellular vesicles (EVs) collected from failing hearts of transverse aortic constriction (TAC) treated mice [28]. **miR-199a-3p** was shown to have protective potential against ischemia-reperfusion injury, increasing cell viability and survival [29,30]. Exogenous administration of miR-199a stimulates cardiomyocyte proliferation, restores functional parameters post-myocardial infarction in mice by inducing cell cycle re-entry in adult cardiomyocytes [31] and induces cardiac regeneration in neonatal mouse cardiomyocytes in vitro and in vivo [32]. **miR- 342-5p** overexpression attenuates ischemia/reperfusion injury in mouse model by targeting and suppressing GPRC5A, reducing cell apoptosis [33]. **miR-625-5p** has little data on post-MI remodeling. MiR-625 inhibits the CAKII/STAT3 pathway, attenuating myocardial hypertrophy [34–36].

**2. Negative Regulators (Downregulated in Post-MI)**

**MiR-15b-5p** and **miR-361-5p** are suppressed in adaptive responses, exacerbating injury. Restoring these miRNAs could counteract apoptotic and inflammatory pathways post-MI.

**miR- 15b-5p** affects cell apoptosis by regulating the expression of Arl2 and Bcl2 proteins. Research by Hullinger et al. showed that miR-15b expression was induced in cardiomyocytes under hypoxic conditions and further increased under conditions of subsequent reperfusion and affected cell viability negatively [37]. At the molecular level, it was explored that **miR-361** through inhibition of prohibitin 1 (PHB1) axis contributes to elevated mitochondrial fission and myocardial injury size after ischemia [38] and suppresses EPC activities in CAD patients via targeting VEGF [39]. **MiR-361-5p** may also serve as a biomarker of acute MI and MACE occurrence. In patients with MI examined 3 hours after symptoms onset, expression of miR-361-5p was significantly higher compared to patients with stable coronary artery disease (CAD) and its accumulation was positively associated with the occurrence of major adverse cardiovascular events (MACE) at 3-days. In view of our results, miR-361-5p may be a biomarker of cardiac injury, which was greater in our LVRR group at baseline compared to LVSV but was reversed as a result of post-MI treatment and cardiac rehabilitation, which was not evaluated by Zhang et al. [40].

**3. Mixed-Regulation miRNAs (Context-Dependent Roles)**

**MiR-24-3p**, **miR-29c-3p, miR-127-3p**, **miR-146a-5p** and **miR-429** display dual roles and highlight the complexity of post-MI signaling networks.

Mir 24-3p has been extensively studied in crucial processes of cardiac remodeling including apoptosis, fibrosis and angiogenesis. Overexpression of **miR-24** has been shown to inhibit **apoptosis** in two ways: in vitro and in vivo in the murine MI model by targeting Bim (a proapoptotic protein of the Bcl-2 family) [41] and in vitro in murine cardiomyocytes subjected to ischemia/reperfusion injury by activating the Nrf2-Keap1 pathway [42]. Moreover, it was inversely correlated with HIF-1a- one of the hypoxia-induced factors which are potent regulators of oxygen homeostasis [43]. Overexpression of miR-24 is also beneficial in the context of post-MI **fibrosis**. In vivo, miR-24 could improve cardiac function and attenuate fibrosis two weeks after MI by targeting the furin-TGF-β1 pathway [44]. Conversely, in the context of **neovascularization** post- MI, miR-24 limits endothelial capillary formation in vitro by targeting the GATA2, PAK4 [45] and eNOS [46]. Inhibition of endothelial miR-24 limited myocardial infarct size in mice, had pro-angiogenic effects, and increased endothelial cell survival.

In vitro, **miR-29c** improved cardiac contractility by acting on SERCA2a, but the results were not confirmed in an in vivo MI model [47].. Downregulation of miR-29 after MI may also contribute to TGFβ-regulated post-MI fibrosis, and raising miR expression levels may be an effective way to reduce the extent of adverse cardiac remodeling [48]. MiR-29a exhibit distinct time-dependent expression profiles that correlate with left ventricular remodeling phases after MI, an increase in miR-29a at 5 days post MI was related to greater adverse remodeling 3 months post MI. **[**49**].** On the other hand, overexpression of miR-29 promoted apoptosis in an ischemia/reperfusion rat model. In vivo, administration of miR-29a or miR-29c antagomirs significantly reduced infarct size and apoptosis via increased expression of Mcl-2, an anti-apoptotic protein of the Bcl-2 family [50].

**MiR-127-3p** was found to be upregulated in human carotid atherosclerotic plaque and expressed in macrophages. It leads to unfavorable changes in fatty acids metabolism and decreases plaque stability. In an in vitro model miR-127-3p expression was positively associated with macrophage accumulation and increased proliferation [51]. Another study based on a murine model of cardiac fibrotic tissue examined 3 weeks after MI showed that miR-127-3p is consistently upregulated in fibrotic tissue and that subsequent forced upregulation of miR-127-3p stimulated further proliferation and fibrosis [52]

On the other hand, miR-127-3p was consistently downregulated in serum samples of 113 MI patients as well as in vitro, in a model of hypoxia and reoxygenation treated cardiomyocytes. Cell transfection was used to overexpress miR127-3p, and it was shown to attenuate apoptosis and exert anti-inflammatory effects via targeting cyclin-dependent kinase inhibitor 3 (CDKN3). However, unlike the aforementioned study, the time point at which the samples were tested was not provided. In addition, details of invasive and pharmacological treatment for MI patients were not included, and the control group consisted of patients with no cardiovascular disease burden [53].

Such results in the context of our observations may indicate that miR-127-3p may not be a biomarker of reverse remodeling due to its high expression, but rather its expression dynamics at 8 weeks post-infarction is an indicator of the remodeling processes that are occurring. In our study, miR-127-3p expression was higher in the LVRR group at baseline and decreased after 8 weeks of follow-up, whereas the expression level changed relatively less in the SLVS group. This may indicate the extent to which the regulation of the complex processes involved in cardiac remodeling must alter to allow cardiac regeneration after myocardial infarction.

**MiR-146a** was shown to inhibit the Fos-AP1 pathway which then results in matrix metalloproteinase 9 (MMP-9) activity downregulation. Metalloproteinases suppression counteracts ventricular remodeling, myocardial dysfunction and development of heart failure [54]. A similar underlying mechanism of miR-146a action was presented in a study by Dong et al. They suggested that an increase in MLCK3 and p-MLC2 through a decrease in MMP2/16 by miR-146a-5p is a compensatory mechanism that may protect the contractile function of the heart after myocardial infarction. Interestingly, aging impaired this miR-146a-5p regulated axis, which resulted in cardiac dysfunction and increased susceptibility to heart failure [55]. Overexpression of miR- 146 was also shown to have a negative effect in post-MI patients – in two studies, higher levels of miR- 146a were associated with a higher proportion of MACE occurrence in patients after ST-segment elevation myocardial infarction [56,57].

**MiR-429** regulates the response to hypoxia due to its association with HIF-1a [55]. Researchers suggest that the action of this miR- is context-dependent – its elevated expression is invaluable in the acute phase, balancing the response to hypoxia by suppressing HIF1a and preventing excessive angiogenesis and inflammation [56–59]. **MiR-429-3p** was shown to be downregulated in cardiac fibrosis models. It was identified that circNSD1, which acts as a sponge to inhibit miR-429-3p, leads to activation of the SULF1/Wnt/β-catenin axis and promotes collagen production in human cardiac fibroblasts [60,61]. In other contexts, miR-429 downregulation alleviated apoptosis and protected cardiomyocytes against hypoxia-induced apoptosis through Notch1 [62].

**4. miRNAs in Other Cardiovascular Diseases**

**MiR-18b-5p, miR-502-5p** and **miR-376c-3p** are involved in non-MI pathology but may overlap with post-MI pathways

**MiR-18b-5p** serves as a marker of HF mortality; its decrease after 48h of hospitalization for acute heart failure (AHF) was found to be predictive for 180-day mortality [63]. In a meta-analysis of mRNA transcription profiles from human blood samples, **miR-502-5p** was one of the down regulated miRNAs in stable coronary artery disease [64], but was upregulated in sera of chronic heart failure (CHF) patients compared to healthy controls [65].

### Pathway enrichment analysis

In the next step of our exploratory analysis, we performed GO enrichment analysis to investigate the functional roles of the predicted target genes of the identified candidate miRNA biomarkers. MiRNAs with high biomarker potential were analyzed to determine the signaling pathways in which they target genes participate. This investigation revealed that the top enriched processes were associated with signaling pathways involved in the process of regulating metabolism of fatty acids, glycolysis, and ECs, such as PI3K-Akt, FoxO, MAPK, HIF-1 pathways, highlighting the biological relevance of our observations (Figs 6 and 7) [66–69]. Network analysis showed that the genes are shared among the associated processes, further supporting the central importance of those pathways.

**Fig 7:**
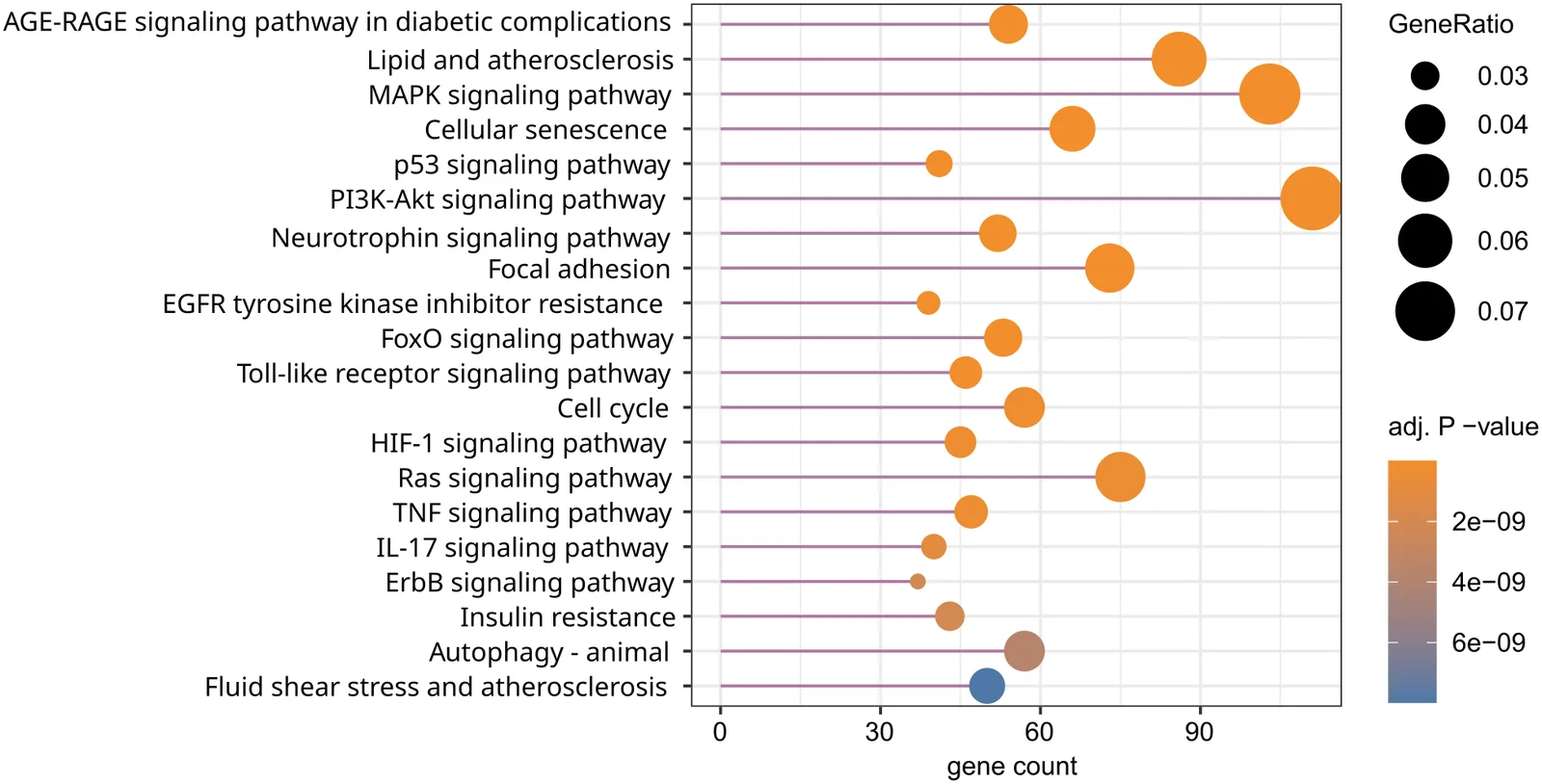
Enrichment analysis of pathways from Kyoto Encyclopedia of Genes and Genomes (KEGG) database.

The analysis of pathways enrichment performed on the union of all targets predicted to interact with 14 miRNAs being high potential markers against the KEGG database.

The size of nodes represents the gene ratio that refers to the ratio of enriched genes to all target genes. The colors represent adjusted *P-*values, that reflects the statistical significance of the enrichment. The higher the *P*-value, the closer the color is to blue. The P-values were calculated with hypergeometric test and corrected for multiple testing with Benjamini-Hochberg method.

Our findings support current literature indicating that these pathways play a key role in cardiac remodeling processes after MI, encompassing processes such as apoptosis [70], hypertrophy [71,72], fibrosis [73], inflammation, ischemia/reperfusion injury [74]. Regulation of these signaling pathways by miRNAs may provide a mechanistic basis for reverse cardiac remodeling [75].

The pathways identified in our analysis, including MAPK, PI3K-AKT, and FOXO, are among the well-established mechanisms of LV reverse remodeling (LVRR) reported in preclinical and clinical studies. While specific biological mechanisms have been suggested to guide remodeling processes, it is also important to move beyond the analysis of individual miRNA-target interactions and adopt a systemic-level perspective.

Predicted interactions between our candidate miRNAs and these signaling pathways suggest a potential mechanistic basis for left ventricular reverse remodeling. However, it is important to note that our pathway enrichment findings are based on in silico predictions. While these bioinformatic tools generate valuable hypotheses regarding the biological relevance of the identified miRNAs in cellular stress responses, extracellular vesicle communication, and fatty acid metabolism, they do not prove direct causal mechanisms. Extensive in vitro and in vivo experimental validation is necessary to confirm whether these candidate miRNAs functionally regulate the PI3K-Akt, FoxO, and MAPK pathways in post-MI recovery.

One can speculate that if a miRNA is associated with a better response to the rehabilitation after MI, its target genes may also play a role in the rehabilitation process, as a miRNA exert their functions through interactions with target genes. To test this hypothesis, we also performed analysis on the lists of genes predicted to interact with miRNAs of high biomarker potential. The in-depth analysis of miRNA: target interactions revealed several genes that can act as hub genes interacting with miRNAs from the candidate biomarker set (Fig 5). Among them genes with the highest number of interactions are two genes with gene activation functions (*ZZZ3* and *SP1*). ZZZ3 (*Zinc Finger ZZ-Type Containing 3*) is a component of ATAC complex, required for maintenance of histone acetylation and gene activation and participated in numerous processes, including regulation of cell division. Whereas SP1 is a zinc finger transcription factor involved in a wide range of cellular processes, including cell differentiation, cell growth, apoptosis and immune responses. Among the hub genes, we also observed genes that participate in key signaling pathways that, when inhibited, may support the healing processes.

Our results have potential clinical implications. Beyond preclinical studies, miRNAs are extensively studied in clinical settings as potential therapeutic molecules across fields such as oncology, dermatology, neurology, or cardiology [76]. For example, an antagomir of miR-132- a synthetic analog of miRNA that functions as a silencing agent- is currently being studied in a Phase 2 trial in post-myocardial infarction patients for its potential to improve systolic function and inhibit myocardial fibrosis processes [77]. Among the miRNAs we identified as potential candidate biomarkers for reverse remodeling, a miR-29 analog is being tested as an inhibitor of skin fibrosis (Phase 2 completed; NCT03601052). Deficiency of miR-29 promotes fibrotic processes not only in the heart but also in the skin, lungs, and corneas [78]. The miR-29 analog is expected to inhibit the excessive synthesis of collagen and other extracellular matrix components [79].

In addition to miRNAs, other non-coding RNA fragments are worth mentioning, such as small interfering RNAs (siRNAs). Inclisiran is currently used in clinical practice to treat hypercholesterolemia [80], and patisiran is used to treat transthyretin amyloidosis [81].

### Study limitations

Although the present results clearly support the involvement of miRNAs in cardiac remodeling, and the pathway enrichment results are consistent with the existing literature on the molecular mechanisms of cardiac remodeling, several limitations of our study should be acknowledged.

The small sample size (n = 10 per group) is a significant limitation. While this size resulted from our strict echocardiographic selection strategy, which was designed to ensure clinical group homogeneity, performing a discovery screening of 375 circulating miRNAs on such a small cohort inherently elevates the expected false discovery risk. Since the pooled design did not allow for statistical corrections for multiple testing, the likelihood of false-positive results and unreliable estimates is significant. Consequently, we explicitly emphasize the exploratory nature of this study. The 14 identified molecules must not be viewed as validated biomarkers, but strictly as preliminary candidate miRNAs that require robust validation in substantially larger, independent cohorts to confirm their reproducibility and true predictive value.

A methodological limitation of this study is the pooling of patient samples prior to miRNA profiling. While pooling serves as a feasible strategy for preliminary biomarker screening, it reduces biological variability. Consequently, this approach prevents the assessment of inter-individual variability, and direct correlations between miRNA expression levels and specific clinical characteristics. Because reproducibility cannot be assessed within a pooled framework, the 14 candidate miRNAs identified in this study must be interpreted as preliminary signals. Our candidate selection relies on descriptive fold-change cutoffs, which represent a preliminary signal requiring validation at the individual patient level.

Furthermore, a limitation of this pilot study is the lack of an independent validation cohort. Because no external validation was performed, the actual predictive performance, diagnostic accuracy, and clinical utility of the 14 identified candidate miRNAs remain unknown.

From an analytical perspective, the quantification of circulating miRNAs in this study faced technical constraints regarding the assay detection threshold. We applied descriptive filtering criteria based on fold-change and cycle threshold values, utilizing the *minProb* method to perform statistical imputation for any miRNAs whose expression levels fell below the reliable limits of the RT-qPCR assay (Ct ≥ 35). Complete exclusion of miRNAs with missing values was avoided, as excluding left-censored data can introduce selection bias by discarding clinically relevant biomarkers that are specifically induced in response to ischemia. Imputing left-censored values near the minimal background threshold allows for relative fold-change estimates without artificially inflating abundance. Although we hypothesize that the absence of a detectable signal may reflect meaningful biological downregulation or “molecular silence” associated with the termination of specific signaling pathways, this approach restricts absolute quantification at specific time points. We cannot definitively differentiate between a physiological absence and expression at unmeasurably low levels. Future studies utilizing ultra-sensitive molecular techniques, such as droplet digital PCR (ddPCR), are necessary to validate these temporal dynamics.

The SLVS group had significantly better systolic function at baseline, however this was by design. As the study focused on exploring biomarkers of recovery, we aimed to include in the control group post-MI patients receiving the same pharmacological treatment and participating in the same rehabilitation program to eliminate as many confounding factors as possible in assessing differences between groups and parameters that interfere with miRNAs assays, such as antiplatelet drugs.

Discrepancy in baseline LV function introduces a valid confounding possibility: the distinct miRNA profiles observed in the LVRR group might partially reflect greater infarct severity, larger initial myocardial injury, or more profound initial ventricular dysfunction, rather than a capacity for recovery. Due to the pooled design of our miRNA screening phase, it was not feasible to perform statistical adjustments for these baseline echocardiographic parameters. However, the temporal dynamics of the identified miRNAs challenge the assumption that they are solely markers of necrosis. Majority (13 out of 14) of our candidate miRNAs maintained robust, highly regulated expression levels over the two-month period in the LVRR group. This sustained temporal signature points toward a continuous, functionally active epigenetic program orchestrating the reverse remodeling cascade, rather than a transient reflection of the initial ischemic insult. Nonetheless, future studies utilizing individual patient samples must adjust for baseline infarct size and LVEF to definitively isolate the predictive value of these candidate biomarkers.

Finally, a notable limitation of our study design is the inability to isolate the specific impact of cardiac rehabilitation on the observed miRNA profiles. Whether the observed molecular profiles are a direct effect of cardiac rehabilitation or reflect natural processes of myocardial reverse remodeling cannot be conclusively determined. In the present study, we do not consider rehabilitation as a factor modifying miRNA expression, but rather as an important element with documented efficacy in improving clinical outcomes after MI and increasing the likelihood of LVRR. Post-MI cardiac rehabilitation is an EBM-derived standard of care. Therefore, performing a comparative study on the effect of rehabilitation vs. patients treated only pharmacologically would not be ethically correct.

The findings from this pilot study are intended to serve exclusively as hypothesis-generating data and enable an analysis of the predictive potential of miRNAs in a larger validation cohort.

Establishing these non-coding RNAs as reliable clinical biomarkers of left ventricular reverse remodeling will require future prospective validation studies utilizing individual samples in larger, independent populations.

### Clinical Perspectives

This pilot study suggests that circulating microRNA (miRNA) profiles measured within the first 72 hours post-myocardial infarction (MI) may offer early insights into a patient’s potential for left ventricular reverse remodeling (LVRR). Preliminary identification of 14 candidate biomarker points toward a possible method for early patient stratification. While these findings are promising, clinicians should view them as an initial step toward identifying high-risk cohorts who might eventually benefit from intensified clinical surveillance or personalized rehabilitation strategies.

A notable strength of our findings is the translation of preclinical and animal model data to human clinical settings, as we observed the association between peripheral blood miRNA signatures and cardiac remodeling processes in our cohort of post-MI patients undergoing rehabilitation and optimal medical treatment. The enrichment of identified target genes in the PI3K-Akt, MAPK, and FoxO signaling pathways suggests a molecular framework for how these miRNAs might influence cardiac repair. Future research in larger, multi-center validation cohorts is essential to confirm the reproducibility, sensitivity, and predictive accuracy of these biomarkers before they can be considered for integration into clinical diagnostic or therapeutic protocols. We hope that the current research will stimulate further investigation of this important area. The rapid advancement of medical technology and the increasing availability of modern therapies based on non-coding RNAs offer a promising vision for the future management of chronic diseases, including heart failure.

## Supporting information

S1 Table Identification of plasma miRNA biomarkers in patients after myocardial infarction. C1- stable left ventricle status group at baseline; C2- stable left ventricle status group at 8 weeks; R1- left ventricle reverse remodeling group at baseline; R2- left ventricle reverse remodeling group at 8 weeks.
(XLSX)

S2 Table Individual patient-level echocardiographic data of the cohort selected for miRNA profiling. Variables prefixed with ‘0_’ denote baseline measurements obtained during the acute phase (Day 3 post-MI). Variables prefixed with ‘8w_’ denote measurements obtained at the 8-week follow-up. E/e’- ratio of early mitral inflow velocity to early diastolic mitral annular velocity; GLS- global longitudinal strain; LVEDD- left ventricular end-diastolic diameter; LVEDDi- indexed left ventricular end-diastolic diameter; LVEDV- left ventricular end-diastolic volume; LVEF- left ventricular ejection fraction; LVESV- left ventricular end-systolic volume; PALS- peak atrial longitudinal strain; TRV- tricuspid regurgitation velocity.
(XLSX)
