# Supplementary material for: Circulating microRNAs as potential candidate biomarkers of left ventricular reverse remodeling after myocardial infarction: A pilot study
Source: PLoS One. 2026 Oct 15;21(10):e0360414. doi: 10.1371/journal.pone.0360414 (PMC13671575; doi:10.1371/journal.pone.0360414)
Supplement: Supplementary file 1 [file pone.0360414.s004.pdf]

Response to Reviewers

Dear Reza Eshraghi, M.D., Academic Editor, PLOS ONE, Dear Reviewers,

We would like to express our sincere gratitude for the time and effort you dedicated to reviewing our manuscript. We deeply appreciate the insightful and highly relevant comments provided. We have carefully addressed each point, which has significantly improved the methodological clarity, clinical interpretation, and overall quality of our paper.

As requested, we have softened the definitive language throughout the text, explicitly reframing this work as an exploratory pilot study. Furthermore, we have provided detailed clarifications regarding our clinical cohort selection, laboratory protocols, and the temporal dynamics of our molecular findings.

Before proceeding to the point-by-point responses, and in the spirit of full transparency, we would like to highlight a methodological textual refinement we made in the *'miRNA Biomarker Candidate Selection'* section. In the original submission, the text stated that for high-potential miRNAs, "Ct values were not imputed in any of the other samples." We have corrected this to accurately reflect our actual bioinformatic pipeline: Ct values were strictly not imputed in the baseline acute-phase samples (R1 and C1).

This clarification is central to our updated analytical rationale, which we discuss extensively in response to your comments below. While robust, non-imputed quantification was strictly required during the acute phase (Day 3) to confirm the initial release of the candidate biomarker, a late-stage drop below the detection threshold (necessitating imputation at the 8-week follow-up) reflects a meaningful biological silencing of the signal rather than an analytical failure. This textual correction does not alter our final selection of the 14 candidate miRNAs, but it ensures complete internal consistency and provides a crucial biological framework for interpreting the temporal dynamics of our findings.

Please find below our point-by-point responses to the Journal Requirements, Editor’s Comments, and Reviewers’ Comments.

JOURNAL REQUIREMENTS & EDITOR COMMENTS

1. Style requirements and file naming.

Response: We have reviewed the PLOS ONE style templates and adjusted the manuscript formatting, headings, and file names accordingly.

2-5. Funding Statement and Financial Disclosure.

2. Funding information should not appear in the Acknowledgments section or other areas of your manuscript. We will only publish funding information present in the Funding Statement section of the online submission form.

Please remove any funding-related text from the manuscript and let us know how you would like to update your Funding Statement.

Please include your amended statements within your cover letter; we will change the online submission form on your behalf.

Response: As requested by the Editorial Office, we have removed all funding-related text from the Acknowledgments section of the manuscript.

Please update our online Funding Statement on our behalf to read exactly as follows:

"The determination of the miRNA profile was carried out using funds from a Young Investigator Grant awarded by the Medical University of Warsaw **(Grant number: 2W6/1/M/MB/N/23)** MiRNA profiling analyses were performed at the IIMCB Bioinformatics Facility (IN-MOL-CELL Infrastructure) funded by the European Union – NextGenerationEU under the National Recovery and Resilience Plan. The IN-MOL-CELL Infrastructure was also funded by the European Union under Horizon Europe (Project 101059801 - RACE) and by the RACE-PRIME project carried out within the IRAP program of the Foundation for Polish Science, co-financed by the European Union under the European Funds for Smart Economy 2021-2027 (FENG)."

3. We note that the grant information you provided in the 'Funding Information' and 'Financial Disclosure' sections do not match.

When you resubmit, please ensure that you provide the correct grant numbers for the awards you received for your study in the 'Funding Information' section.

4. Please state what role the funders took in the study. If the funders had no role, please state: "The funders had no role in study design, data collection and analysis, decision to publish, or preparation of the manuscript."

If this statement is not correct you must amend it as needed.

Please include this amended Role of Funder statement in your cover letter; we will change the online submission form on your behalf.

5. Thank you for stating the following financial disclosure:

"MiRNA profiling analyses were performed at the IIMCB Bioinformatics Facility (IN-MOL-CELL Infrastructure) funded by the European Union – NextGenerationEU under National Recovery and Resilience Plan. The determination of the miRNA profile was carried out using funds from a Young Investigator Grant awarded by the Medical University of Warsaw."

We note that one or more of the authors is affiliated with the funding organization, indicating the funder may have had some role in the design, data collection, analysis or preparation of your manuscript for publication; in other words, the funder played an indirect role through the participation of the co-authors. If the funding organization did not play a role in the study design, data collection and analysis, decision to publish, or preparation of the manuscript and only provided financial support in the form of authors' salaries and/or research materials, please do the following:

-Review your statements relating to the author contributions and ensure you have specifically and accurately indicated the role(s) that these authors had in your study. These amendments should be made in the online form.

- Confirm in your cover letter that you agree with the following statement, and we will change the online submission form on your behalf: "The funder provided support in the form of salaries for authors [insert relevant initials], but did not have any additional role in the study design, data collection and analysis, decision to publish, or preparation of the manuscript. The specific roles of these authors are articulated in the 'author contributions' section.

Response: Regarding the Role of Funder (points 4 and 5 from your request), we would like to clarify that the funding organization (Medical University of Warsaw) did not provide authors'

salaries through this specific grant. The grant was utilized strictly for research materials and laboratory analyses.

Therefore, we respectfully amend the requested disclosure statement to accurately reflect this:
"The funders provided financial support strictly for research materials and laboratory assays, but did not provide salaries for the authors. The funders had no role in study design, data collection and analysis, decision to publish, or preparation of the manuscript."

6. Figure Resolution.

Response: We have provided new, high-resolution copies of Figures that comply with PLOS ONE figure guidelines.

Reviewer #1:

Major Revisions

1. Reframe Claims of "Statistical Significance" to "Descriptive Differences." The manuscript states that samples were pooled, meaning biological variance cannot be assessed. Despite this, the Abstract claims miRNAs were identified with "significantly higher baseline accumulation," and the limitations section states, "We applied stringent statistical analysis". Relying on descriptive fold-change and Ct thresholds is not a "stringent statistical analysis," nor does it yield "significant" results in a statistical sense. Please systematically review the manuscript and remove terms like "significantly" and "stringent statistical analysis" when referring to the pooled miRNA expression data. Replace them with accurate descriptive terminology."

2. Tone Down Definitive Conclusions. The Conclusion explicitly states, "This study identifies 14 miRNAs as LVRR biomarkers in post-MI cardiac regeneration". While the descriptive thresholds used, like Fold Change and Ct, are perfectly acceptable for an exploratory pilot study generating candidates, they are not rigorous enough to definitively "identify" these molecules as validated biomarkers. Revise the conclusions in the Abstract and the main text to explicitly reflect the exploratory nature of the study. Adjust the language to state that the study identifies 14 candidate

miRNAs that serve as potential LVRR biomarkers requiring further validation in larger, non-pooled cohorts.

Response: We completely agree with the Reviewer. Since the miRNA screening was performed on pooled samples, the lack of biological variance precludes formal statistical significance testing. We have systematically reviewed the entire manuscript (including the Title, Abstract, Results, and Conclusion) and removed definitive terms such as "significantly," "stringent statistical analysis," and "identifies 14 miRNAs as LVRR biomarkers." We have reframed the language to explicitly state that the study identifies 14 *candidate* miRNAs based on descriptive fold-change thresholds, representing preliminary signals that require further validation in independent, individual-level cohorts.

3. Clarify Contradiction in Patient Matching. The Methods state that the SLVS patients were "matched by age, sex, comorbidities" to the LVRR group. Table 3 shows a stark difference in sex distribution: the LVRR group is 50% male, while the SLVS group is 90% male. This raises concern that the groups were not adequately matched by sex. Please correct this discrepancy. Either remove the claim that the groups were matched by sex in the Methods or explain the limitation/reasoning behind this gender imbalance.

Response: Due to the limited pool of patients unambiguously meeting these extremes, matching was primarily based on age, comorbidities, and standard medical therapy rather than exact gender parity, which we now explicitly state as a limitation. To provide full transparency, we have added a detailed Study Flow Diagram (**new Figure 1**) and updated the Materials and Methods section to explicitly describe the disposition of all 39 patients.

4. Justify the Imputation of Missing Data on Pooled Samples. The methodology notes that 107 out of 375 miRNAs were not quantified in any sample and that missing values were imputed using the minProb method. While minProb is a standard bioinformatic tool, applying statistical imputation to a single pooled dataset may introduce additional uncertainty and potential bias,

because a missing value here represents an entire cohort of 10 patients rather than a single technical failure or individual patient outlier. Please expand your Discussion or Limitations section to explicitly justify why this imputation method was appropriate for pooled samples and acknowledge how it might bias the candidate selection compared to simply excluding unquantified miRNAs.

Response:

We thank the Reviewer for this perceptive comment regarding data processing in pooled RT-qPCR experiments. We agree that missing values in pooled samples represent a cohort-wide signal falling below the limit of detection (LOD) rather than an individual technical outlier. Below, we detail our rationale for using the minProb method, how potential bias was mitigated in our selection pipeline, and how we have updated the Limitations section:

- Rationale for minProb vs. complete case analysis

In RT-qPCR profiling, missing data primarily result from non-detection due to ultra-low circulating concentration (left-censored missingness, Non-Random / MNAR) rather than random technical failures. Excluding any miRNA with missing values across samples would create a severe selection bias. Specifically, it would eliminate candidate biomarkers that are strongly induced/upregulated in disease or recovery states (e.g., detectable at baseline in LVRR group, R1, but below the detection threshold in stable controls, C1). Imputing non-detects near the limit of detection (via Gaussian distribution centered on minimal values) preserves the ability to identify dynamic off-to-on biological switches

- Mitigation of imputation bias in candidate biomarker selection

We recognize that imputing pooled samples introduces statistical uncertainty regarding individual patient variability within the pool. To directly prevent imputed values from driving false-positive discoveries, we implemented a multi-tiered stratification framework for biomarker candidates (High, Mid, Low potential) based on assay reliability and imputation status. Crucially, robust, non-imputed empirical quantification was strictly required at the acute baseline phase (Day 3; pools R1 and C1) for all 14 miRNAs identified as "high potential" candidates. The vast majority of these candidates (11 out of 14) were fully quantified across the entire dataset with zero imputed values at any time point. For the remaining 3 candidates, imputation was permitted *exclusively* at the 8-

week follow-up. Rather than viewing this as a technical artifact, we hypothesize that this downregulation below the detection threshold reflects an active "molecular silence" tied to the physiological termination of early signaling pathways. Consequently, the core findings, hub gene networks, and functional pathways reported in this study are grounded in robust acute-phase expression and are safeguarded against baseline imputation-derived bias.

Manuscript Changes:

- We have expanded the Limitations section to explicitly discuss the statistical impact of imputing left-censored data in pooled assays and how our selection strategy minimizes candidate selection bias
- In Materials and Methods (Subsection: miRNA Profiling) we changed the text explaining the left-censored nature of missing qPCR values, defining minProb as a probabilistic algorithm centered on minimal quantified intensity to preserve low-abundance biological gradients without inflating fold changes
- In the Discussion we added information on the statistical trade-offs of imputing left-censored data in pooled cohorts and how our selection framework safeguarded the study against imputation artifacts.

5. Clarify Patient Enrollment and Attrition The text states that 39 patients were enrolled based on the inclusion/exclusion criteria in Table 1, but only 20 were selected for final analysis. Table 1 only lists baseline entry criteria, which all 39 patients presumably passed. The manuscript does not explain the clinical outcomes or disposition of the remaining 19 patients, leaving a significant gap in the reader's understanding of the cohort selection. Please add a study flow diagram to visually clarify the selection process and document the disposition of all 39 initially enrolled patients.

Response: We thank the Reviewer for highlighting this omission. Out of the 39 patients initially enrolled, all 39 successfully completed the full 4-week cardiac rehabilitation program and the 8-week echocardiographic follow-up. However, to maximize the molecular signal-to-noise ratio in our pooled miRNA analysis, we decided to profile two distinct clinical groups from this cohort:

patients demonstrating clear left ventricular reverse remodeling (LVRR, n=10) and a reference group of patients whose echocardiographic parameters remained relatively stable without significant structural improvement over the observation period (SLVS, n=10).

The remaining 19 patients were excluded from the molecular profiling phase because they exhibited intermediate echocardiographic trajectories (e.g., LVEF improvement of 5–8%) that neither met the criteria required for the LVRR group, nor the strict stability required for the SLVS group.

Manuscript Changes:

To provide full transparency, we have added a detailed Study Flow Diagram (new Figure 1) and updated the Materials and Methods section to explicitly describe the disposition of all 39 patients.

<u>Minor Revisions</u>

1. Critical Typographical Error Regarding Ct Thresholds: In the Methods section under miRNA Profiling, the text states: "filtered to remove all undetected or unreliable (with Ct < 35) miRNAs". The mathematical symbol is inverted. Removing Ct values less than 35 would discard the highly expressed, reliable data. This also contradicts later sentences, which correctly identify unreliable miRNAs as having a "Ct > 35". Please correct the symbol in this sentence to accurately reflect your methodology.

Response: We sincerely thank the Reviewer for pointing out this critical typographical error. You are entirely correct—the inequality sign was inadvertently inverted in that sentence, which contradicted both our intended methodology and the subsequent descriptions in the text. We confirm that highly expressed, reliable miRNAs ($C_t < 35$) were retained, while unquantified or unreliable miRNAs ($C_t \geq 35$) were filtered out/imputed as near the limit of detection. We have corrected the inequality sign in the revised manuscript to accurately reflect our data filtering criteria.

2. Troponin Unit Inconsistency: In Table 2, the unit for "cTnI 1st" is given as "ng/l", but "cTnI 2nd" is given as "ng/ml". Given the reported values (median 12,902 for cTnI 2nd), "ng/ml" is highly likely a typo. Please verify and standardize the units.

Response: Thank you for highlighting the inconsistency. The troponin unit in Table 2 has been corrected to ng/l.

3. Numerical Discrepancy Regarding Assayed miRNAs: There is an inconsistency regarding the number of miRNAs analyzed. The Abstract, Introduction, and Methods sections repeatedly state that the expression of 380 miRNAs was quantified. However, the Results section states: "Of the 375 miRNAs represented on the plate, 107 were not quantified". Please verify the exact number of miRNAs on the array and ensure this number is consistent throughout the entire manuscript.

Response: We thank the Reviewer for highlighting this inconsistency. We apologize for the confusion caused by the conflicting counts in the text. After re-verifying the layout of the RT-qPCR array panel: The array contains a total of 384 wells, which includes 380 target miRNAs alongside 4 internal controls (spike-in controls: 363, ath-miR159a, cel-miR-39-3p, and cel-miR-39-3p). Additionally, the array features technical replicates for internal reference for intra-plate normalization and calibration: hsa-miR-16-5, hsa-miR-221-3p and hsa-miR-450b-5p. Therefore, the actual number of unique human microRNAs assayed was 375, while 380 represented the total number of reaction wells per plate for target miRNAs. We apologize for the initial confusion caused by interchanging target miRNA reaction wells (380) with unique target miRNAs (375). We have updated the entire manuscript to explicitly clarify this distinction and ensure absolute consistency throughout. The numerical discrepancy regarding assayed miRNAs has been standardized throughout the text

4. Standardize the "PALS" / "LA strain" Terminology: The manuscript uses inconsistent terminology for this parameter. Table 4 uses "LA strain" for baseline but switches to "PALS" for 8 weeks. In the Methods, it is defined as both "peak left atrial strain" and "Peak atrial longitudinal strain". In the footnotes for Tables 4 and 5, it is written as "left atrium peaks longitudinal strain",

which contains a grammatical error. Please standardize this to a single, correct expansion consistently throughout the text and tables.

Response: We have standardized the echocardiographic terminology to PALS (peak atrial longitudinal strain) throughout the entire manuscript, tables, and footnotes, and clarified in the Methods that it reflects left atrial reservoir function.

5.Reference Formatting Errors: Reference 23 is corrupted, as it mistakenly includes the title and authors of Reference 22 ("et al. igraph: Network Analysis and Visualization in R").

6. Missing Spaces: Correct formatting typos such as "additional10 miRNAs" and "R1sample".

Response: Reference formatting errors and missing spaces have been corrected.

Reviewer #2:

Major Comments

1. The manuscript overstates the biomarker and regeneration claims The central conclusion that the identified miRNAs represent biomarkers of cardiac regeneration after MI is not sufficiently supported by the presented data. The study demonstrates an association between circulating miRNA profiles and subsequent LVRR; however, it does not demonstrate: • predictive accuracy, • diagnostic performance, • clinical utility, • causal involvement in myocardial regeneration. The term "cardiac regeneration" is also problematic because improvement in ventricular function after MI represents reverse remodeling rather than confirmed regeneration of myocardial tissue. I strongly recommend revising the terminology throughout the manuscript. The title, abstract, and conclusions should be modified to reflect that these are candidate miRNAs associated with LVRR rather than validated biomarkers of regeneration.

Response: We have replaced the term "cardiac regeneration" with "reverse remodeling" (LVRR) throughout the manuscript, including the Title. We have also explicitly rephrased our conclusions

to state that these are "candidate biomarkers" associated with LVRR, and we emphasize that diagnostic performance and clinical utility remain unknown pending external validation.

2. Major concern regarding sample size and statistical power. The study includes only a very limited number of patients, with 10 patients in the LVRR group and 10 patients in the SLVS group. A discovery analysis involving hundreds of miRNAs requires substantially larger sample sizes to avoid: • false-positive findings, • unstable estimates, • overfitting, • poor reproducibility.

The manuscript identifies 14 candidate miRNAs; however, given the exploratory nature and limited cohort size, these findings should be interpreted cautiously. The authors should: • explicitly state that the study is exploratory, • avoid definitive biomarker language, • provide a justification for sample size, • discuss the expected false discovery risk.

Response:

We highly appreciate this critical and constructive concern. We fully agree with the Reviewer that a sample size of 10 patients per group in a high-throughput screening of 375 miRNAs inherently carries elevated risks of false-positive discoveries.

To fully address your concerns, we have fundamentally adjusted the tone of the manuscript and expanded the methodological transparency.

Manuscript Changes:

- **Explicit statement of exploratory nature & avoidance of definitive language:** We have reviewed the manuscript to eliminate any definitive biomarker language. We now consistently use terms such as "candidate biomarkers," "preliminary signals," and "hypothesis-generating data." We explicitly emphasize that this is a pilot/exploratory study.
- **Justification for sample size:** We have clarified our sample size rationale in the Methods and Limitations sections. Out of the 39 patients who completed the full rehabilitation and follow-up program, we employed an "extreme phenotype" selection strategy. We selected 10 patients demonstrating the most pronounced reverse remodeling (LVRR) and 10 reference patients whose parameters remained relatively stable without significant structural improvement (SLVS). While this deliberate selection reduced the

sample size, it was designed to maximize clinical group purity and molecular contrast (signal-to-noise ratio) for the pooled discovery phase.

- **Statistical implications and false discovery risk:** Furthermore, the pooled design necessitated by this cohort size inherently precludes standard variance-based statistical testing (such as p-values or False Discovery Rate correction). We have thoroughly addressed this specific statistical limitation, along with our candidate selection thresholds and data imputation strategy, in our detailed response to Comment 5 below. We have also expanded the Study Limitations section to explicitly discuss the expected false discovery risk.

3. The use of pooled samples. The most important methodological concern is the pooling of patient samples before miRNA analysis. Pooling reduces biological variability and prevents evaluation of individual patient-level differences. Although pooling may be used for preliminary screening, it prevents: • statistical inference at the patient level, • assessment of variability, • correlation with clinical characteristics, • reproducibility assessment. The manuscript should clarify: • exactly how samples were pooled, • whether equal volumes or equal RNA amounts were used, • how technical variability was controlled. The authors should clearly state that the findings represent preliminary signals requiring validation in individual patient samples.

Response: We completely agree with the Reviewer's assessment. While pooling is a practical strategy for an initial exploratory screening, we acknowledge that it eliminates biological variability, precluding statistical inference at the individual patient level, and direct correlation with individual clinical characteristics.

To address your concerns, we have made the following revisions:

- **Pooling Methodology:** We have updated the Materials and Method*s* section to explicitly state that samples were pooled using equal volumes of plasma from each of the 10 patients per group prior to RNA extraction, ensuring uniform contribution to the pooled sample.

- **Technical Variability Control:** As detailed in our previous responses and the revised manuscript, technical variability was minimized by pre-analytical processing

(simultaneous aliquoting, uniform centrifugation to prevent platelet contamination), eliminating batch effects (all RNA isolations and RT-qPCR runs were performed simultaneously), and applying geometric mean (geomean) normalization of stable miRNAs to adjust for any minor technical fluctuations.

- **Preliminary Nature of the Findings:** We have revised the Study Limitations and Conclusions sections to state explicitly that our results represent preliminary, hypothesis-generating signals. We clearly emphasize that validation in larger, independent cohorts using individual patient samples is absolutely required to establish clinical utility.

4. Lack of independent validation cohort. A biomarker discovery study requires validation in an independent population. At present, the manuscript identifies candidate miRNAs but does not demonstrate whether these markers can reliably predict LVRR. The authors should acknowledge that: • no external validation was performed, • predictive performance remains unknown. If possible, validation using individual samples from an independent cohort would significantly strengthen the manuscript.

Response: We sincerely appreciate the Reviewer's critical assessment and fully agree with this limitation. As this was designed as an exploratory pilot study, its primary objective was hypothesis generation and the identification of candidate targets, rather than definitive biomarker validation.

We acknowledge that we did not perform external validation using an independent cohort. Consequently, the true predictive performance, diagnostic accuracy, and clinical utility of the identified 14 candidate miRNAs currently remain unknown. While we completely agree that validation using individual samples from an independent population would significantly strengthen the study, such an extensive validation phase falls outside the scope and resources of the present pilot investigation.

Manuscript Changes:

To ensure complete transparency, we have explicitly added these caveats to the Study Limitations section, clearly stating that no external validation was performed and that these findings must be interpreted strictly as preliminary signals requiring future validation.

5. Statistical analysis requires substantial clarification. The manuscript analyzes a large number of miRNAs; however, the statistical approach requires more detail.

Major concerns:

Multiple testing correction. The manuscript does not clearly demonstrate correction for multiple comparisons. For hundreds of tested miRNAs, false discovery rate control is essential. The authors should provide: • adjusted p-values, • FDR methodology, • justification for candidate selection thresholds.

Imputation strategy. Missing miRNA values were imputed; however, with such a small dataset, imputation can substantially influence results. The authors should provide: • the percentage of missing values, • sensitivity analyses, • justification for the selected imputation method.

Response: We thank the Reviewer for raising these statistical considerations. We welcome the opportunity to clarify the analytical framework of our study and the methodological rationale behind candidate selection and data imputation.

1. **Multiple testing correction and candidate selection thresholds**

   A) Absence of *P*-values / *q*-values

   Because this exploratory screening phase evaluated pooled serum samples (representing pooled cohorts across conditions) without individual sample replicates per pool, standard inferential statistical tests (such as t-tests or Mann-Whitney U test) and downstream false discovery rate corrections cannot be calculated. Inferential statistics require within-group variance, which is absent when comparing single pooled measurements.

   B) Justification for Selection Thresholds

   To compensate for the lack of variance-based testing during this initial discovery phase, we applied a multi-tier filtering protocol designed to minimize false-positive biomarker discovery:

- high-potential candidates: fold change ≥ 8.0 (equivalent to a ΔCt ≥ 3.0 between groups) and a minimal normalized abundance in the R1 group corresponding to normalized Ct ≥ 10

  Rationale: Represents high-amplitude regulatory shifts paired with robust, unambiguous baseline expression in the clinical response group
- mid-potential candidates: fold change ≥ 5.0 (equivalent to a ΔCt ≥ 2.32 between groups) and a minimal normalized abundance in the R1 group corresponding to normalized Ct ≥ 5

  Rationale: Captures substantial differential expression with moderate baseline intensity in responders.
- low-potential candidates:fold change ≥ 5.0 (equivalent to a ΔCt ≥ 2.32 between groups) and a minimal normalized abundance in the R1 group corresponding to normalized Ct ≥ 5

  Rationale: Identifies targets with noteworthy relative expression shifts but assigns a lower priority tier due to low baseline signal intensity near background noise thresholds

2. Imputation strategy and sensitivity analysis

A) The percentage of missing values

Across the array of 375 unique target miRNAs profiled in 4 experimental pools (375 × 4 = 1,500 total potential data points):

- 28.5% (107miRNAs, 428 data points) were completely undetected in all four experimental pools (0/4 detection)
- 29.6% (111 miRNAs, 255 data points) were partially detected, requiring imputation for the missing condition(s)
- 41.9% (157 miRNAs, 628 data points) were quantified across all four experimental pools (4/4 detection) with zero missing or imputed values

B) Sensitivity considerations in pooled screening

In pooled RT-qPCR profiling, combining 10 patient samples dilutes low-abundance miRNAs by a factor of 10, theoretically delaying detection by approximately 3.3 cycles ($\Delta Ct = \log_2(10)$). Consequently, missing data points in our pooled dataset primarily reflect

trace-level miRNAs that fell just below the assay's Limit of Detection (LOD) due to sample dilution, rather than random technical failures

C) Justification for minProb

In RT-qPCR profiling, missing data primarily result from non-detection due to ultra-low circulating concentration (left-censored missingness, Non-Random / MNAR) rather than random technical failures. Excluding any miRNA with missing values across samples would create a severe selection bias. Specifically, it would eliminate candidate biomarkers that are strongly induced/upregulated in disease or recovery states (e.g., detectable at baseline in LVRR responders, R1, but below the detection threshold in stable controls, C1). Imputing non-detects near the limit of detection (via Gaussian distribution centered on minimal values) preserves the ability to identify dynamic off-to-on biological switches.

Manuscript Changes:

- we have expanded the Limitations section to explicitly discuss the statistical impact of imputing left-censored data in pooled assays and how our selection strategy minimizes candidate selection bias
- in Materials and Methods (Subsection: miRNA Profiling) we changed the text explaining the left-censored nature of missing qPCR values, defining minProb as a probabilistic algorithm centered on minimal quantified intensity to preserve low-abundance biological gradients without inflating fold changes
- in the Discussion we added information on the statistical trade-offs of imputing left-censored data in pooled cohorts and how our tiered selection framework successfully safeguarded the study against imputation artifacts

5.Baseline differences between groups may confound interpretation. The LVRR and SLVS groups differed significantly in baseline cardiac function. The SLVS group had better baseline EF and GLS. This raises the possibility that observed miRNA differences reflect:

- infarct severity,
- myocardial injury extent,
- initial ventricular dysfunction, rather than true predictors of recovery.

The authors should discuss this limitation more extensively. If possible, adjustment for baseline echocardiographic parameters should be considered.

Response: We agree that the baseline differences in left ventricular function (e.g., lower LVEF and worse GLS in the LVRR group) introduce a potential confounding factor, raising the valid question of whether the observed miRNA signatures merely reflect greater initial infarct severity. Due to pooling, we cannot perform multivariable regression to adjust for this.

Prompted by the Reviewers' insightful comments regarding the potential confounding effect of baseline infarct severity and the risk of passive molecular leakage, we have fundamentally deepened our analysis of the temporal miRNA expression dynamics.

As now detailed in our Discussion, an in-depth analysis of data imputation patterns allowed us to significantly expand our interpretation of miRNA temporal behavior. Notably, 11 out of 14 candidates showed robust, non-imputed expression across all time points in the LVRR group, contradicting the expectation of a rapid, uniform post-necrosis decay. More importantly, only 3 out of 14 miRNAs required imputation. For two of them (miR-18b-5p and miR-342-5p), imputation was required *only* in the SLVS group at 8 weeks, while their signal remained firmly detectable in the LVRR group. This asymmetrical downregulation contradicts the theory of a simple, universal fading of necrotic markers. Instead, we hypothesize that these specific pro-survival pathways naturally extinguish in patients whose left ventricular morphology remains stable (SLVS), whereas they are actively maintained in patients undergoing structural reverse remodeling (LVRR). Finally, miR-429 demonstrated a highly specific, acute "on/off" burst, fading in both groups by week 8.

We believe that highlighting these distinct temporal trajectories—framed cautiously as hypothesis-generating observations—provides a logical counterargument to the passive leakage theory and elevates the biological relevance of our manuscript.

7. Pathway analysis should be interpreted more cautiously. The pathway enrichment analysis provides interesting hypotheses; however, predicted miRNA-target interactions and enrichment analyses do not establish biological mechanisms. Statements suggesting that identified miRNAs regulate: • regeneration, • repair, • recovery pathways, should be softened. The manuscript should clearly distinguish: • predicted biological relevance, • experimentally validated mechanisms.

Response: We thank the Reviewer for this important methodological reminder. To address this issue, we have carefully reviewed and softened the language surrounding the bioinformatic predictions. We now explicitly use terms such as "predicted targets," "hypothetical interactions," and "potential regulatory roles," and we state in the Discussion that these *in silico* hypotheses require rigorous experimental validation.

8.Clarify the relationship between rehabilitation and miRNA changes. The study population received cardiac rehabilitation. However, the design cannot determine whether: • rehabilitation influenced miRNA expression, • miRNA profiles simply reflected spontaneous post-MI recovery, • miRNA differences represented baseline injury severity. This should be clarified.

Response: Because cardiac rehabilitation (CR) is an evidence-based standard of care, it would be ethically unacceptable to include a non-rehabilitation control group. Consequently, we cannot definitively decouple the impact of CR from spontaneous post-MI recovery. We have added a dedicated paragraph in the *Study Limitations* to clarify that our candidate miRNAs should be viewed as predictors of overall favorable clinical trajectories under optimal medical and rehabilitative care, rather than specific biomarkers of exercise response.

Minor Comments

1. The manuscript should consistently use "candidate biomarkers" instead of "biomarkers."
Response: We completely agree with this important terminological distinction. We have carefully reviewed the entire manuscript (including the Title, Abstract, Results, and Discussion) and systematically replaced the word "biomarkers" with "candidate biomarkers" to accurately reflect the preliminary, hypothesis-generating nature of our discovery-phase study.

2. The term "regeneration" should be replaced with "reverse remodeling" unless direct regeneration measurements are available.

Response: We thank the Reviewer for pointing out this imprecision. Since our study assessed functional and structural cardiac recovery via macroscopic echocardiographic parameters rather than direct cellular regeneration or cardiomyocyte proliferation, we have removed the term "regeneration" throughout the text. It has been replaced with the more accurate and clinically appropriate terms "reverse remodeling" or "myocardial recovery."

3. Provide more detailed demographic and clinical characteristics

Response: We appreciate this suggestion and agree that a thorough clinical profile is essential. We would like to politely direct the Reviewer to **Tables 2 and 3** in the manuscript, which provide a highly comprehensive breakdown of over 25 distinct demographic and clinical variables. This includes detailed metrics such as Body Mass Index (BMI), physical capacity (METs), precise ischemia-reperfusion times, infarct-related artery (IRA) distribution, extensive lipid and glycemic profiles, cardiac biomarkers (NT-proBNP, cTnI), and a complete pharmacological profile. We believe these tables comprehensively characterize our pilot cohort. To ensure maximum clarity, we have meticulously reviewed these sections to verify that all relevant clinical data collected during the study is fully and transparently reported.

4.Include individual patient-level echocardiographic changes if possible.

Response: We agree that transparency regarding patient-level clinical data provides valuable granularity. Accordingly, we have compiled the individual echocardiographic trajectories (including exact baseline and 8-week follow-up measurements for LVEF, ESV, and EDV) for all included patients. This data is now provided in the manuscript as a new Supplementary Table 2.

5. Report exact statistical values rather than only significance thresholds.

Response: We thank the Reviewer for highlighting this. While exact *p*-values were provided for all comparisons within our tables, we acknowledge that a generic significance threshold ($p > 0.05$) was inadvertently left in the main text of the *Results* section when describing pharmacotherapy differences. We have corrected this by replacing the threshold with the exact statistical values for each medication class (e.g., $p = 0.53$ for ACE inhibitors, $p = 0.07$ for SGLT2 inhibitors) to ensure full compliance with the journal's reporting guidelines.

6. Clarify miRNA normalization procedures

Response: We have revised the **Materials and Methods (miRNA Profiling)** section to provide a concise and clear explanation of our bioinformatic pipeline.

Raw Ct values were first filtered to exclude unreliable signals (Ct ≥ 35). To remove non-specific inter-sample variation, we applied Global Mean Normalization. A sample-specific normalization factor was calculated as the geometric mean of all fully quantified miRNAs. Normalized expression levels (ΔCt) were derived by subtracting individual target Ct values from this mean (ΔCt = Ct_mean - Ct_target). For convenient interpretation, these values were transformed to a positive scale (proportional to log2 copy numbers) by subtracting them from a constant size factor, and relative fold changes were calculated using the 2^-ΔΔCt method.

Furthermore, we clarified our handling of missing values. We applied the minProb imputation method. This probabilistic algorithm models missing values using a Gaussian distribution centered on the minimal quantified intensity. This approach prevents the severe selection bias of complete-case analysis and preserves the biological gradients of low-abundance miRNAs without artifactually inflating fold-change calculations.

7.Provide additional information regarding technical replicates

Response: We have added necessary clarifications regarding the technical setup in the Materials and Methods section. Due to the standardized, multiplexed nature of the 384-well TaqMan® Advanced miRNA Human A Card, the microfluidic design does not include technical replicates for each of the 380 individual miRNAs on a single card. The array features technical replicates for internal reference for intra-plate normalization and calibration: hsa-miR-16-5, hsa-miR-221-3p and hsa-miR-450b-5p. This limitation has also been transparently noted in the Study Limitations section.

8. Improve explanation of miRNA selection criteria

Response: We have revised the **miRNA Profiling and miRNA Biomarker Candidate Selection** sections to precisely reflect our bioinformatic pipeline. We clarified that to compensate for the lack of variance-based testing on pooled samples, we applied a multi-tier filtering protocol (High,

Mid, and Low potential) based on descriptive fold-change cutoffs combined with a requirement for a highly reliable baseline signal (minimal normalized Ct > 10 for high-potential candidates). Most importantly, we explicitly stated that candidates were only prioritized as "high-potential" if their Ct values were strictly *not imputed* in the baseline acute-phase samples (Day 3; pools R1 and C1).

9. The discussion should better address alternative explanations for the findings

Response: We are grateful for this constructive comment. Prompted by this feedback, we have substantially expanded the Discussion to address competing hypotheses—most notably, the alternative explanation that elevated baseline miRNA levels in the LVRR group might simply be a passive reflection of a larger initial infarct size and extensive necrosis, given the discrepancy in baseline LVEF.

10. Avoid implying therapeutic potential without experimental validation.

Response: We have carefully reviewed the Discussion and Conclusions to soften our language and eliminate any speculative implications regarding direct therapeutic applications. We explicitly stated that extensive in vitro and in vivo experimental validation is required to definitively confirm the functional role of these candidate miRNAs in cardiac recovery before any therapeutic or clinical diagnostic potential can be considered.

11. Suggested update of references The authors are encouraged to update some of the older references in the Introduction and Discussion with more recent reviews that better reflect the current understanding of non-coding RNA biology in cardiovascular disease. Several cited studies provide important foundational knowledge; however, newer literature on extracellular vesicle-associated miRNAs and broader non-coding RNA regulation of myocardial injury and remodeling would improve the scientific context of the manuscript. In particular, the authors may consider replacing some older general references on miRNA involvement in cardiovascular disease with more recent reviews discussing the role of circulating/exosomal miRNAs and regulatory RNA networks in myocardial infarction and cardiac remodeling.

A few of suggested references:

1. https://doi.org/10.3390/life16030400

2. https://doi.org/10.3390/ijms27031488

3. https://doi.org/10.1016/j.prp.2024.155274

4. https://doi.org/10.3389/fcvm.2024.1429858

Response: We are grateful for the recommendation to update our bibliography. We completely agree that the field of non-coding RNAs is evolving rapidly, and incorporating recent literature—especially studies focusing on extracellular vesicle-associated miRNAs and temporal dynamics—greatly enhances the scientific context of our manuscript.

We have carefully reviewed the excellent literature provided and incorporated updates into the revised text:

- **In the Introduction:** We have removed the older general reference (former Ref 12) and replaced it with recent, comprehensive insights into exosomal miRNAs and broader non-coding RNA regulatory networks in cardiac remodeling. Specifically, we incorporated https://doi.org/10.3390/life16030400 and https://doi.org/10.3389/fcvm.2024.1429858.
- **In the Discussion:** We enriched our analysis of time-dependent miRNA expression by incorporating the suggested preclinical experimental evidence **[**https://doi.org/10.3390/ijms27031488 into our newly formulated subsection on the *Temporal dynamics of miRNA expression*.

While we deeply appreciate all the provided literature suggestions (including 10.1016/j.prp.2024.155274), we selected those that most directly align with our study's specific focus on circulating temporal miRNA trajectories. This selective approach allowed us to thoroughly modernize our theoretical background while maintaining a concise and strictly relevant reference list. Thank you for directing our attention to these valuable resources.

Reviewer #3:

1. Thirty-nine patients were recruited, but only 20 were selected for miRNA analysis. The manuscript does not adequately explain the exclusion of the other 19 participants or the matching

procedure. Furthermore, the LVRR group had significantly worse baseline LVEF and GLS than the stable group. Patients with poorer baseline function naturally have more opportunity to improve, creating regression to the mean. The observed miRNA differences may therefore reflect greater initial myocardial injury rather than a biological capacity for regeneration.

Response: Please see our detailed responses to Reviewer #1 regarding patient attrition (all 39 patients finished rehabilitation; 19 intermediate phenotypes were excluded to ensure group purity, now visualized in **Figure 1**) and to Reviewer #2 regarding baseline differences. We have expanded the Discussion to address the regression to the mean hypothesis, utilizing our temporal expression data to argue that the sustained miRNA levels at week 8 reflect active reverse remodeling rather than passive leakage proportional to baseline injury severity.

2. The manuscript would benefit from a clearer and more thorough comparison of its findings with prior human studies investigating circulating non-coding RNAs and post-myocardial infarction remodeling. In particular, attention was drawn to several relevant works, including a review (PMID 39171328) discussing MALAT1 and broader non-coding RNA mechanisms in myocardial infarction, an earlier study (PMID 21956146) that explored temporal plasma miRNA profiles in relation to left ventricular remodeling after MI, and a more recent prospective study (PMID 38256409) involving 80 patients, which did not find a significant predictive association between selected circulating miRNAs and post-MI remodeling.

Response: We are grateful to the Reviewer for directing us to these highly relevant clinical studies. We completely agree that thoroughly contrasting our findings with prior human trials provides crucial context and highlights the methodological nuances of circulating biomarker research.

- PMID 21956146 (Temporal profiles): We utilized this foundational clinical study to broaden our section on the miR-29 family in the Discussion. By referencing their findings on distinct time-dependent expression profiles, we were able to explain the seemingly dual/mixed roles of these miRNAs in the literature, which supports our own hypothesis regarding the temporal dynamics of reverse remodeling.

- PMID 39171328 (MALAT1 and broader ncRNA mechanisms): We expanded our Introduction to discuss how miRNAs do not operate in isolation but function within broader, highly complex epigenetic networks.

3. There are important inconsistencies in the molecular methods. The manuscript states in one section that miRNAs with Ct <35 were removed, while elsewhere Ct >35 is described as unreliable. This must be corrected.

Response: We thank the Reviewer for highlighting this typographical error. You are entirely correct, the inequality sign was inadvertently inverted in that sentence, which created a contradiction with the rest of the text. We confirm that highly expressed, reliable miRNAs (Ct < 35) were retained for analysis, whereas unquantified or unreliable miRNAs (Ct ≥ 35) were filtered out and treated, as near the limit of detection. We have corrected the mathematical symbol in the revised manuscript to ensure complete consistency throughout the text.

4.The use of minProb imputation, originally developed mainly for proteomic data, is insufficiently justified for pooled RT-qPCR measurements. Imputation may artificially produce large fold changes when a miRNA is undetected in one pool.

Response: We thank the Reviewer for this important and insightful comment regarding the application of minProb to pooled RT-qPCR profiling. We welcome the opportunity to justify our methodological rationale and clarify how our candidate selection pipeline explicitly safeguarded against artificial fold-change inflation.

While minProb was originally introduced in mass-spectrometry proteomics, its statistical foundation applies directly to high-throughput RT-qPCR profiling. Both technologies generate non-random, left-censored missing data (Missing Not At Random / MNAR), where missingness is caused by target concentrations falling below the assay's Limit of Detection (LOD) rather than random technical failures. Imputing missing values with a random draw from a Gaussian distribution centered on minimal detected intensities provides a realistic lower-bound estimate near

the background threshold. This prevents complete-case analysis from discarding biologically vital off-to-on switches (targets induced from below LOD in controls to robust expression in responders).

We fully agree with the Reviewer that imputing an undetected value in one pool while comparing it to a detected value in another can artifactually drive large relative fold changes (ΔCt).

To prevent technical artifacts from skewing candidate selection while preserving meaningful biological data, we applied a highly specific, time-dependent filtering strategy.

Robust, non-imputed empirical quantification (Ct < 35) was strictly required at the baseline acute phase (Day 3; pools R1 and C1) to confirm the initial, reliable release of the biomarker. Candidate prioritization required not only high relative fold changes but also robust baseline expression in the target responder group (R1) (normalized Ct > 10 for high-potential candidates and normalized Ct > 5 for mid-potential candidates). Low-abundance targets near the background signal (normalized Ct < 5) at baseline were downgraded to the low-potential tier.

Consequently, among the 14 high-potential candidate miRNAs subjected to downstream functional analysis, the vast majority (11 out of 14) possessed 100% complete empirical quantification across all four experimental pools (C1, C2, R1, and R2) with zero imputed values. For the remaining 3 candidates, data imputation was allowed strictly at the 8-week follow-up. Rather than viewing this as a technical failure or assay error, we hypothesize that this drop below the reliable detection threshold (necessitating minProb imputation) may reflect a meaningful biological downregulation. Missing values in high-throughput RT-qPCR profiling typically represent non-random, left-censored data, where microRNA abundance falls below the assay's Limit of Detection (LOD), rather than random technical failures. We interpret this as an active "molecular silence" associated with the termination of specific early signaling pathways once the structural rescue is initiated.

Because robust quantification was strictly mandatory at the critical Day 3 baseline, our candidate selection effectively minimized technical bias while allowing us to capture profound biological silencing at the late-stage follow-up.

Manuscript Changes:

- we have expanded the Limitations section to explicitly discuss the statistical impact of imputing left-censored data in pooled assays and how our selection strategy minimizes candidate selection bias

- in Materials and Methods (Subsection: miRNA Profiling) we changed the text explaining the left-censored nature of missing qPCR values, defining minProb as a probabilistic algorithm centered on minimal quantified intensity to preserve low-abundance biological gradients without inflating fold changes
- in the Discussion we added information on the statistical trade-offs of imputing left-censored data in pooled cohorts and how our tiered selection framework safeguarded the study against imputation artifacts

5. The candidate thresholds, including fold change ≥8 or ≥5, appear arbitrary and are not supported by formal differential-expression analysis or false-discovery-rate correction.

Response: We thank the Reviewer for these constructive comments. We welcome the opportunity to clarify our normalization methodology, detail our multi-step biomarker discovery framework, and explain how global expression correlation analysis was used to support candidate selection.

1. **Threshold Defense**

Because initial screening was conducted on pooled serum cohorts representing distinct clinical groups without individual sample replicates per pool, standard inferential statistical tests (such as t-tests or Mann-Whitney U test) could not be calculated due to the lack of within-group variance. To ensure that candidate selection was statistically sound and biologically driven, we utilized a structured, multi-tier filtering pipeline starting with global correlation analysis. Following data normalization and baseline imputation, we compared global miRNA expression profiles across experimental groups. Pearson correlation analysis demonstrated high consistency between control group timepoints ($r = 0.80$), whereas correlations between rehabilitation responders and controls at both day 3 ($r = 0.67$) and week 8 ($r = 0.75$) were markedly lower. This confirmed distinct, system-wide expression shifts driven by clinical status rather than random technical noise. Given that acute myocardial infarction induces systemic miRNA release, we focused on miRNAs upregulated in responders relative to controls. To prioritize candidates for downstream target prediction, the upregulated miRNAs were stratified into operational confidence tiers anchored to clear cycle threshold shifts (ΔCt). While chosen cutoffs may seem arbitrary, anchoring them to specific cycle shifts ensured a transparent and straightforward selection process. Specifically, a 5-

fold shift ($\Delta Ct \geq 2.32$) captures pronounced differential expression beyond a standard 4-fold (2.0 Ct) benchmark, while an 8-fold shift ($\Delta Ct \geq 3.0$) represents an order-of-magnitude biological induction that far exceeds the typical technical noise threshold of RT-qPCR assays (approx. 0.5-1 Ct). When combined with global correlation trends mentioned above, these thresholds may provide a robust framework for prioritizing candidate biomarkers.

Manuscript Changes:

- in Materials and Methods (Subsection: miRNA Profiling) we added text explaining why the differential expression could not be performed and we underlined significance of Pearson correlation in our analysis

2. Normalization Strategy

Data normalization was performed using the Global Mean Normalization method, which is established as an optimal approach for high-throughput qPCR panels.

Specifically, raw Ct values were baseline-corrected, and unquantified targets were flagged ($Ct \geq 35$). For each sample pool, the mean Ct of all fully quantified targets ($Ct < 35$) was calculated to establish a sample-specific normalization factor (Ct_{mean}). Normalized target levels (ΔCt) were derived by subtracting the individual target Ct from Ct_{mean} ($\Delta Ct = Ct_{mean} - Ct_{target}$). Relative fold changes between groups were subsequently determined using the $2^{-\Delta\Delta Ct}$ method.

Manuscript Changes:

- in Materials and Methods (Subsection: miRNA Profiling) we

reviewed text explaining the normalization method to clearly explain this methodology

6. The normalization method, hemolysis assessment, platelet contamination, technical replicates, and batch effects also require clarification

Response: We thank the Reviewer for pointing out the need for a more detailed pre-analytical description. We have substantially expanded the Materials and Methods (Subsection: Plasma samples). Specifically:

- **Platelets & Hemolysis:** Blood was centrifuged at 5000 rpm for 10 min at 4°C. Plasma was aspirated by keeping the pipette tip strictly below the surface and leaving the boundary layer (buffy coat) intact to prevent platelet/leukocyte contamination. Visual inspection was

performed, and hemolyzed samples were discarded. The stability of *miR-16-5p* (our endogenous control) further confirmed the absence of significant hemolysis.

- **Batch Effects & Technical Replicates:** To eliminate batch effects, equal volumes of plasma from each patient were pooled, and all samples were aliquoted, frozen at -80°C, and then thawed and processed simultaneously in a single experimental RNA isolation and RT-qPCR run. While technical replicates were not feasible on the highly multiplexed TaqMan Array Cards due to budget constraints, absolute quantities were robustly normalized using the geometric mean (geomean) of all stable assayed miRNAs.

7. The study measures short-term echocardiographic reverse remodeling, not direct cardiac regeneration. Improved LVEF may result from recovery of myocardial stunning, successful reperfusion, pharmacological treatment, loading-condition changes, or measurement variability. Similarly, because all patients received rehabilitation and there was no comparison group without rehabilitation, the authors cannot conclude that the miRNAs predict "response to cardiac rehabilitation."

Response: We fully agree. We have removed the term "regeneration" and replaced it with "reverse remodeling" (LVRR) throughout the manuscript. We have also added a limitation stating that we cannot differentiate the isolated effect of rehabilitation from spontaneous recovery or standard pharmacological treatment.

We hope that these extensive revisions fully address the concerns raised and that our manuscript is now acceptable for publication in PLOS ONE.
