# Supplementary material for: Circulating microRNAs as potential candidate biomarkers of left ventricular reverse remodeling after myocardial infarction: A pilot study
Source: PLoS One. 2026 Oct 15;21(10):e0360414. doi: 10.1371/journal.pone.0360414 (PMC13671575; doi:10.1371/journal.pone.0360414)
Supplement: Peer review history

# Peer review: Decision Letter 0

**DOI:** 10.1371/journal.pone.0360414.r001

**Authors:** Reza Eshraghi

25 Jun 2026

Dear Dr. Klimczak-Tomaniak,

Thank you for submitting your manuscript to PLOS ONE. After careful consideration, we feel that it has merit but does not fully meet PLOS ONE’s publication criteria as it currently stands. Therefore, we invite you to submit a revised version of the manuscript that addresses the points raised during the review process.

Please submit your revised manuscript by Aug 24 2026 11:59PM. If you will need more time than this to complete your revisions, please reply to this message or contact the journal office at plosone@plos.org. When you're ready to submit your revision, log on to https://www.editorialmanager.com/pone/ and select the 'Submissions Needing Revision' folder to locate your manuscript file.

- A letter that responds to each point raised by the academic editor and reviewer(s). You should upload this letter as a separate file labeled 'Response to Reviewers'.
- A marked-up copy of your manuscript that highlights changes made to the original version. You should upload this as a separate file labeled 'Revised Manuscript with Track Changes'.
- An unmarked version of your revised paper without tracked changes. You should upload this as a separate file labeled 'Manuscript'.

If you would like to make changes to your financial disclosure, please include your updated statement in your cover letter. Guidelines for resubmitting your figure files are available below the reviewer comments at the end of this letter.

If applicable, we recommend that you deposit your laboratory protocols in protocols.io to enhance the reproducibility of your results. Protocols.io assigns your protocol its own identifier (DOI) so that it can be cited independently in the future. For instructions see: https://journals.plos.org/plosone/s/submission-guidelines#loc-laboratory-protocols. Additionally, PLOS ONE offers an option for publishing peer-reviewed Lab Protocol articles, which describe protocols hosted on protocols.io. Read more information on sharing protocols at https://plos.org/protocols?utm_medium=editorial-email&utm_source=authorletters&utm_campaign=protocols.

As the corresponding author, your ORCID iD is verified in the submission system and will appear in the published article. PLOS supports the use of ORCID, and we encourage all coauthors to register for an ORCID iD and use it as well. Please encourage your coauthors to verify their ORCID iD within the submission system before final acceptance, as unverified ORCID iDs will not appear in the published article. *Only*  the individual author can complete the verification step; PLOS staff *cannot*  verify ORCID iDs on behalf of authors.

We look forward to receiving your revised manuscript.

Kind regards,

Reza Eshraghi, M.D

Academic Editor

PLOS One

Journal Requirements:

When submitting your revision, we need you to address these additional requirements.

1. Please ensure that your manuscript meets PLOS ONE's style requirements, including those for file naming. The PLOS ONE style templates can be found at

https://journals.plos.org/plosone/s/file?id=wjVg/PLOSOne_formatting_sample_main_body.pdf and

https://journals.plos.org/plosone/s/file?id=ba62/PLOSOne_formatting_sample_title_authors_affiliations.pdf

2. Thank you for stating the following in the Acknowledgments Section of your manuscript:

“MiRNA profiling analyses were performed at the IIMCB Bioinformatics Facility (IN-MOL-CELL Infrastructure) funded by the European Union – NextGenerationEU under National Recovery and Resilience Plan. IN-MOL-CELL Infrastructure was also funded by the European Union under Horizon Europe (Project 101059801 - RACE) and by RACE-PRIME project carried out within the IRAP program of the Foundation for Polish Science co-financed by the European Union under the European Funds for Smart Economy 2021-2027 (FENG).”

We note that you have provided funding information that is not currently declared in your Funding Statement. However, funding information should not appear in the Acknowledgments section or other areas of your manuscript. We will only publish funding information present in the Funding Statement section of the online submission form.

Please remove any funding-related text from the manuscript and let us know how you would like to update your Funding Statement. Currently, your Funding Statement reads as follows:

“MiRNA profiling analyses were performed at the IIMCB Bioinformatics Facility (IN-MOL-CELL Infrastructure) funded by the European Union – NextGenerationEU under National Recovery and Resilience Plan. The determination of the miRNA profile was carried out using funds from a Young Investigator Grant awarded by the Medical University of Warsaw.”

Please include your amended statements within your cover letter; we will change the online submission form on your behalf.

3. We note that the grant information you provided in the ‘Funding Information’ and ‘Financial Disclosure’ sections do not match.

When you resubmit, please ensure that you provide the correct grant numbers for the awards you received for your study in the ‘Funding Information’ section.

4. Thank you for stating the following financial disclosure:

“MiRNA profiling analyses were performed at the IIMCB Bioinformatics Facility (IN-MOL-CELL Infrastructure) funded by the European Union – NextGenerationEU under National Recovery and Resilience Plan. The determination of the miRNA profile was carried out using funds from a Young Investigator Grant awarded by the Medical University of Warsaw.”

Please state what role the funders took in the study.  If the funders had no role, please state: "The funders had no role in study design, data collection and analysis, decision to publish, or preparation of the manuscript."

If this statement is not correct you must amend it as needed.

Please include this amended Role of Funder statement in your cover letter; we will change the online submission form on your behalf.

5. Thank you for stating the following financial disclosure:

“MiRNA profiling analyses were performed at the IIMCB Bioinformatics Facility (IN-MOL-CELL Infrastructure) funded by the European Union – NextGenerationEU under National Recovery and Resilience Plan. The determination of the miRNA profile was carried out using funds from a Young Investigator Grant awarded by the Medical University of Warsaw.”

We note that one or more of the authors is affiliated with the funding organization, indicating the funder may have had some role in the design, data collection, analysis or preparation of your manuscript for publication; in other words, the funder played an indirect role through the participation of the co-authors. If the funding organization did not play a role in the study design, data collection and analysis, decision to publish, or preparation of the manuscript and only provided financial support in the form of authors' salaries and/or research materials, please do the following:

1. Review your statements relating to the author contributions, and ensure you have specifically and accurately indicated the role(s) that these authors had in your study. These amendments should be made in the online form.

2. Confirm in your cover letter that you agree with the following statement, and we will change the online submission form on your behalf:

“The funder provided support in the form of salaries for authors [insert relevant initials], but did not have any additional role in the study design, data collection and analysis, decision to publish, or preparation of the manuscript. The specific roles of these authors are articulated in the ‘author contributions’ section.

6. Please upload a new copy of Figures 2, 3, 4, 5 and 6 as the detail is not clear. Please follow the link for more information:  https://journals.plos.org/plosone/s/figures

7. If the reviewer comments include a recommendation to cite specific previously published works, please review and evaluate these publications to determine whether they are relevant and should be cited. There is no requirement to cite these works unless the editor has indicated otherwise.

Additional Editor Comments:

Thank you for submitting your manuscript to PLOS ONE.

After careful consideration, we invite you to revise your paper, carefully addressing the comments from the reviewers and the editor. Please ensure the results are accurately reported, any overstated conclusions are rewritten and the limitations of the work fully explained.

In the response letter, please provide sufficient data addressing either the altered text or the reason for not applying reviewers' comments. In addition, the suggested articles by the reviewers are completely optional, and the authors should take the most appropriate and up-to-date references. When your revision is ready, please submit the updated manuscript and a point-by-point response. This will help us move to a swift decision.

We recommend submitting all revisions within the mentioned deadline.

[Note: HTML markup is below. Please do not edit.]

Reviewers' comments:

Reviewer's Responses to Questions

**Comments to the Author**

1. Is the manuscript technically sound, and do the data support the conclusions?

Reviewer #1: Partly

Reviewer #2: Partly

Reviewer #3: Partly

**********

2. Has the statistical analysis been performed appropriately and rigorously?

Reviewer #1: No

Reviewer #2: No

Reviewer #3: Yes

**********

3. Have the authors made all data underlying the findings in their manuscript fully available?

The PLOS Data policy

Reviewer #1: Yes

Reviewer #2: No

Reviewer #3: No

**********

4. Is the manuscript presented in an intelligible fashion and written in standard English?

Reviewer #1: Yes

Reviewer #2: Yes

Reviewer #3: Yes

**********

Reviewer #1: The authors have designed a clinically rigorous and interesting pilot study investigating the role of circulating miRNAs in left ventricular reverse remodeling (LVRR) post-myocardial infarction. The clinical framework of the study is a notable strength. The authors have established clear echocardiographic criteria for LVRR and utilized a highly appropriate control group (SLVS) to effectively isolate the variables of interest.

However, there is a fundamental mismatch between the study’s design (a pilot study utilizing pooled samples) and the definitive language used in the core conclusions. Because the samples were pooled, the molecular data lacks the biological variance necessary for true statistical significance testing. Additionally, there is a contradiction in the text regarding patient matching criteria. Consequently, the manuscript requires major revisions to reframe the findings from definitive "identifications" to exploratory "hypotheses" and to clarify clinical discrepancies.

Please address the following major and minor concerns to ensure the manuscript meets the rigorous reporting standards of PLOS ONE.

Major Revisions (Methodological Framing and Clinical Discrepancies)

1. Reframe Claims of "Statistical Significance" to "Descriptive Differences."

The manuscript states that samples were pooled, meaning biological variance cannot be assessed. Despite this, the Abstract claims miRNAs were identified with "significantly higher baseline accumulation," and the limitations section states, "We applied stringent statistical analysis". Relying on descriptive fold-change and Ct thresholds is not a "stringent statistical analysis," nor does it yield "significant" results in a statistical sense. Please systematically review the manuscript and remove terms like "significantly" and "stringent statistical analysis" when referring to the pooled miRNA expression data. Replace them with accurate descriptive terminology."

2. Tone Down Definitive Conclusions

The Conclusion explicitly states, "This study identifies 14 miRNAs as LVRR biomarkers in post-MI cardiac regeneration". While the descriptive thresholds used, like Fold Change and Ct, are perfectly acceptable for an exploratory pilot study generating candidates, they are not rigorous enough to definitively "identify" these molecules as validated biomarkers. Revise the conclusions in the Abstract and the main text to explicitly reflect the exploratory nature of the study. Adjust the language to state that the study identifies 14 candidate miRNAs that serve as potential LVRR biomarkers requiring further validation in larger, non-pooled cohorts.

3. Clarify Contradiction in Patient Matching

The Methods state that the SLVS patients were "matched by age, sex, comorbidities" to the LVRR group. Table 3 shows a stark difference in sex distribution: the LVRR group is 50% male, while the SLVS group is 90% male. This raises concern that the groups were not adequately matched by sex. Please correct this discrepancy. Either remove the claim that the groups were matched by sex in the Methods, or explain the limitation/reasoning behind this gender imbalance.

4. Justify the Imputation of Missing Data on Pooled Samples

The methodology notes that 107 out of 375 miRNAs were not quantified in any sample and that missing values were imputed using the minProb method. While minProb is a standard bioinformatic tool, applying statistical imputation to a single pooled dataset may introduce additional uncertainty and potential bias, because a missing value here represents an entire cohort of 10 patients rather than a single technical failure or individual patient outlier. Please expand your Discussion or Limitations section to explicitly justify why this imputation method was appropriate for pooled samples, and acknowledge how it might bias the candidate selection compared to simply excluding unquantified miRNAs.

5. Clarify Patient Enrollment and Attrition

The text states that 39 patients were enrolled based on the inclusion/exclusion criteria in Table 1, but only 20 were selected for final analysis. Table 1 only lists baseline entry criteria, which all 39 patients presumably passed. The manuscript does not explain the clinical outcomes or disposition of the remaining 19 patients, leaving a significant gap in the reader's understanding of the cohort selection. Please add a study flow diagram to visually clarify the selection process and document the disposition of all 39 initially enrolled patients.

Minor Revisions

Please address the following minor typographical and formatting errors prior to publication:

1. Critical Typographical Error Regarding Ct Thresholds: In the Methods section under miRNA Profiling, the text states: "filtered to remove all undetected or unreliable (with Ct < 35) miRNAs". The mathematical symbol is inverted. Removing Ct values less than 35 would discard the highly expressed, reliable data. This also contradicts later sentences, which correctly identify unreliable miRNAs as having a "Ct > 35". Please correct the symbol in this sentence to accurately reflect your methodology.

2. Troponin Unit Inconsistency: In Table 2, the unit for "cTnI 1st" is given as "ng/l", but "cTnI 2nd" is given as "ng/ml". Given the reported values (median 12,902 for cTnI 2nd), "ng/ml" is highly likely a typo. Please verify and standardize the units.

3. Numerical Discrepancy Regarding Assayed miRNAs: There is an inconsistency regarding the number of miRNAs analyzed. The Abstract, Introduction, and Methods sections repeatedly state that the expression of 380 miRNAs was quantified. However, the Results section states: "Of the 375 miRNAs represented on the plate, 107 were not quantified". Please verify the exact number of miRNAs on the array and ensure this number is consistent throughout the entire manuscript.

4. Standardize the "PALS" / "LA strain" Terminology: The manuscript uses inconsistent terminology for this parameter. Table 4 uses "LA strain" for baseline but switches to "PALS" for 8 weeks. In the Methods, it is defined as both "peak left atrial strain" and "Peak atrial longitudinal strain". In the footnotes for Tables 4 and 5, it is written as "left atrium peaks longitudinal strain", which contains a grammatical error. Please standardize this to a single, correct expansion consistently throughout the text and tables.

5. Reference Formatting Errors: Reference 23 is corrupted, as it mistakenly includes the title and authors of Reference 22 ("et al. igraph: Network Analysis and Visualization in R").

6. Missing Spaces: Correct formatting typos such as "additional10 miRNAs" and "R1sample".

Reviewer #2: Overall Assessment

This manuscript investigates the potential role of circulating microRNAs as early predictors of left ventricular reverse remodeling (LVRR) following myocardial infarction (MI). The authors performed circulating miRNA profiling in post-MI patients undergoing standard medical therapy and cardiac rehabilitation and identified a panel of 14 candidate miRNAs associated with subsequent improvement in ventricular structure and function. The study further explored predicted target genes and biological pathways associated with these miRNAs.

The topic is clinically important, as early identification of patients likely to experience favorable or unfavorable myocardial recovery after MI remains an unmet need. The integration of echocardiographic phenotyping with molecular profiling represents an interesting translational approach. The manuscript is generally well written, and the authors provide a thoughtful biological interpretation of their findings.

However, the current study has substantial methodological limitations that prevent the conclusions from being supported at the level required for a biomarker discovery study. The major concerns relate to the very small sample size, pooled sample analysis, lack of independent validation, insufficient statistical correction for multiple testing, and overinterpretation of exploratory findings as clinically meaningful biomarkers.

In its current form, I recommend major revision. The manuscript could become valuable as a hypothesis-generating pilot study if the conclusions are appropriately moderated and methodological limitations are more clearly addressed.

Major Comments

1. The manuscript overstates the biomarker and regeneration claims

The central conclusion that the identified miRNAs represent biomarkers of cardiac regeneration after MI is not sufficiently supported by the presented data.

The study demonstrates an association between circulating miRNA profiles and subsequent LVRR; however, it does not demonstrate:

• predictive accuracy,

• diagnostic performance,

• clinical utility,

• causal involvement in myocardial regeneration.

The term “cardiac regeneration” is also problematic because improvement in ventricular function after MI represents reverse remodeling rather than confirmed regeneration of myocardial tissue.

I strongly recommend revising the terminology throughout the manuscript. The title, abstract, and conclusions should be modified to reflect that these are candidate miRNAs associated with LVRR rather than validated biomarkers of regeneration.

2. Major concern regarding sample size and statistical power

The study includes only a very limited number of patients, with 10 patients in the LVRR group and 10 patients in the SLVS group.

A discovery analysis involving hundreds of miRNAs requires substantially larger sample sizes to avoid:

• false-positive findings,

• unstable estimates,

• overfitting,

• poor reproducibility.

The manuscript identifies 14 candidate miRNAs; however, given the exploratory nature and limited cohort size, these findings should be interpreted cautiously.

The authors should:

• explicitly state that the study is exploratory,

• avoid definitive biomarker language,

• provide a justification for sample size,

• discuss the expected false discovery risk.

3. The use of pooled samples is a critical methodological limitation

The most important methodological concern is the pooling of patient samples before miRNA analysis.

Pooling reduces biological variability and prevents evaluation of individual patient-level differences. Although pooling may be used for preliminary screening, it prevents:

• statistical inference at the patient level,

• assessment of variability,

• correlation with clinical characteristics,

• reproducibility assessment.

The manuscript should clarify:

• exactly how samples were pooled,

• whether equal volumes or equal RNA amounts were used,

• how technical variability was controlled.

The authors should clearly state that the findings represent preliminary signals requiring validation in individual patient samples.

4. Lack of independent validation cohort

A biomarker discovery study requires validation in an independent population.

At present, the manuscript identifies candidate miRNAs but does not demonstrate whether these markers can reliably predict LVRR.

The authors should acknowledge that:

• no external validation was performed,

• predictive performance remains unknown.

If possible, validation using individual samples from an independent cohort would significantly strengthen the manuscript.

5. Statistical analysis requires substantial clarification

The manuscript analyzes a large number of miRNAs; however, the statistical approach requires more detail.

Major concerns:

Multiple testing correction

The manuscript does not clearly demonstrate correction for multiple comparisons.

For hundreds of tested miRNAs, false discovery rate control is essential.

The authors should provide:

• adjusted p-values,

• FDR methodology,

• justification for candidate selection thresholds.

Imputation strategy

Missing miRNA values were imputed; however, with such a small dataset, imputation can substantially influence results.

The authors should provide:

• the percentage of missing values,

• sensitivity analyses,

• justification for the selected imputation method.

6. Baseline differences between groups may confound interpretation

The LVRR and SLVS groups differed significantly in baseline cardiac function.

The SLVS group had better baseline EF and GLS.

This raises the possibility that observed miRNA differences reflect:

• infarct severity,

• myocardial injury extent,

• initial ventricular dysfunction,

rather than true predictors of recovery.

The authors should discuss this limitation more extensively.

If possible, adjustment for baseline echocardiographic parameters should be considered.

7. Pathway analysis should be interpreted more cautiously

The pathway enrichment analysis provides interesting hypotheses; however, predicted miRNA-target interactions and enrichment analyses do not establish biological mechanisms.

Statements suggesting that identified miRNAs regulate:

• regeneration,

• repair,

• recovery pathways,

should be softened.

The manuscript should clearly distinguish:

• predicted biological relevance,

• experimentally validated mechanisms.

8. Clarify the relationship between rehabilitation and miRNA changes

The study population received cardiac rehabilitation.

However, the design cannot determine whether:

• rehabilitation influenced miRNA expression,

• miRNA profiles simply reflected spontaneous post-MI recovery,

• miRNA differences represented baseline injury severity.

This should be clarified.

Minor Comments

1. The manuscript should consistently use “candidate biomarkers” instead of “biomarkers.”

2. The term “regeneration” should be replaced with “reverse remodeling” unless direct regeneration measurements are available.

3. Provide more detailed demographic and clinical characteristics.

4. Include individual patient-level echocardiographic changes if possible.

5. Report exact statistical values rather than only significance thresholds.

6. Clarify miRNA normalization procedures.

7. Provide additional information regarding technical replicates.

8. Improve explanation of miRNA selection criteria.

9. The discussion should better address alternative explanations for the findings.

10. Avoid implying therapeutic potential without experimental validation.

11. Suggested update of references The authors are encouraged to update some of the older references in the Introduction and Discussion with more recent reviews that better reflect the current understanding of non-coding RNA biology in cardiovascular disease. Several cited studies provide important foundational knowledge; however, newer literature on extracellular vesicle-associated miRNAs and broader non-coding RNA regulation of myocardial injury and remodeling would improve the scientific context of the manuscript. In particular, the authors may consider replacing some older general references on miRNA involvement in cardiovascular disease with more recent reviews discussing the role of circulating/exosomal miRNAs and regulatory RNA networks in myocardial infarction and cardiac remodeling. A few of suggested references: 1. https://doi.org/10.3390/life16030400 2. https://doi.org/10.3390/ijms27031488 3. https://doi.org/10.1016/j.prp.2024.155274 4. https://doi.org/10.3389/fcvm.2024.1429858

Strengths of the Study

Despite these limitations, the manuscript has several strengths:

• Important and clinically relevant research question.

• Integration of molecular and imaging biomarkers.

• Use of clinically characterized MI patients.

• Exploration of biologically plausible miRNA candidates.

• Appropriate acknowledgment of several limitations.

• Potential value as a pilot study generating hypotheses for larger validation studies.

Final Recommendation

Recommendation: Major Revision

The manuscript addresses an interesting and potentially impactful question; however, the current evidence is insufficient to support the conclusion that the identified miRNAs are validated biomarkers of cardiac regeneration or clinical recovery after MI.

The study should be reframed as an exploratory pilot investigation. The major issues regarding sample size, pooled samples, statistical methodology, and interpretation must be addressed before the manuscript can be considered further.

With substantial revision and more cautious interpretation, the work may provide a useful foundation for future validation studies.

Reviewer #3: Dear authors:

I wish to extend my appreciation for the effort and dedication that went into preparing this manuscript. The topic addressed is of clear scientific and clinical relevance, and I recognize the amount of work required to compile and present such a study. In the following sections, I have outlined my detailed comments and concerns for your consideration:

My comments:

- Thirty-nine patients were recruited, but only 20 were selected for miRNA analysis. The manuscript does not adequately explain the exclusion of the other 19 participants or the matching procedure. Furthermore, the LVRR group had significantly worse baseline LVEF and GLS than the stable group. Patients with poorer baseline function naturally have more opportunity to improve, creating regression to the mean. The observed miRNA differences may therefore reflect greater initial myocardial injury rather than a biological capacity for regeneration.

- the manuscript would benefit from a clearer and more thorough comparison of its findings with prior human studies investigating circulating non-coding RNAs and post-myocardial infarction remodeling. In particular, attention was drawn to several relevant works, including a review (PMID 39171328) discussing MALAT1 and broader non-coding RNA mechanisms in myocardial infarction, an earlier study (PMID 21956146) that explored temporal plasma miRNA profiles in relation to left ventricular remodeling after MI, and a more recent prospective study (PMID 38256409) involving 80 patients, which did not find a significant predictive association between selected circulating miRNAs and post-MI remodeling.

- There are important inconsistencies in the molecular methods. The manuscript states in one section that miRNAs with Ct <35 were removed, while elsewhere Ct >35 is described as unreliable. This must be corrected.

- The use of minProb imputation, originally developed mainly for proteomic data, is insufficiently justified for pooled RT-qPCR measurements. Imputation may artificially produce large fold changes when a miRNA is undetected in one pool.

- The candidate thresholds, including fold change ≥8 or ≥5, appear arbitrary and are not supported by formal differential-expression analysis or false-discovery-rate correction. The normalization method, hemolysis assessment, platelet contamination, technical replicates, and batch effects also require clarification

- The study measures short-term echocardiographic reverse remodeling, not direct cardiac regeneration. Improved LVEF may result from recovery of myocardial stunning, successful reperfusion, pharmacological treatment, loading-condition changes, or measurement variability. Similarly, because all patients received rehabilitation and there was no comparison group without rehabilitation, the authors cannot conclude that the miRNAs predict “response to cardiac rehabilitation.”

**********

what does this mean?). If published, this will include your full peer review and any attached files.

If you choose “no”, your identity will remain anonymous but your review may still be made public.

**Do you want your identity to be public for this peer review?**  For information about this choice, including consent withdrawal, please see our Privacy Policy

Reviewer #1: No

Reviewer #2: No

Reviewer #3: No

**********

[NOTE: If reviewer comments were submitted as an attachment file, they will be attached to this email and accessible via the submission site. Please log into your account, locate the manuscript record, and check for the action link "View Attachments". If this link does not appear, there are no attachment files.]

To ensure your figures meet our technical requirements, please review our figure guidelines: https://journals.plos.org/plosone/s/figures

You may also use PLOS’s free figure tool, NAAS, to help you prepare publication quality figures: https://journals.plos.org/plosone/s/figures#loc-tools-for-figure-preparation.

NAAS will assess whether your figures meet our technical requirements by comparing each figure against our figure specifications.

# Author comment: Author response to Decision Letter 1

**DOI:** 10.1371/journal.pone.0360414.r002

3 Sep 2026

Dear Academic Editor and Reviewers,

We would like to express our sincere gratitude for the time and effort you dedicated to reviewing our manuscript. We deeply appreciate your insightful and highly relevant comments, which have significantly improved the methodological clarity and overall quality of our manuscript.

Due to the character count limits of this text box and the comprehensive nature of our point-by-point responses, we have provided our complete "Response to Reviewers" as a separate PDF file. Please refer to the attached document for our detailed answers.

We hope that our extensive revisions fully address the concerns raised.

Sincerely,

The Authors

Attachment Submitted filename: Response to Reviewers.pdf

# Peer review: Decision Letter 1

**DOI:** 10.1371/journal.pone.0360414.r003

**Authors:** Reza Eshraghi

27 Sep 2026

Circulating microRNAs as potential candidate biomarkers of left ventricular reverse remodeling after myocardial infarction: A pilot study

PONE-D-26-25288R1

Dear Dr. Klimczak-Tomaniak,

We’re pleased to inform you that your manuscript has been judged scientifically suitable for publication and will be formally accepted for publication once it meets all outstanding technical requirements.

Within one week, you’ll receive an e-mail detailing the required amendments. When these have been addressed, you’ll receive a formal acceptance letter and your manuscript will be scheduled for publication.

An invoice will be generated when your article is formally accepted. Please note, if your institution has a publishing partnership with PLOS and your article meets the relevant criteria, all or part of your publication costs will be covered. Please make sure your user information is up-to-date by logging into Editorial Manager at Editorial Manager® and clicking the ‘Update My Information' link at the top of the page. For questions related to billing, please contact billing support.

If your institution or institutions have a press office, please notify them about your upcoming paper to help maximize its impact. If they’ll be preparing press materials, please inform our press team as soon as possible -- no later than 48 hours after receiving the formal acceptance. Your manuscript will remain under strict press embargo until 2 pm Eastern Time on the date of publication. For more information, please contact onepress@plos.org.

Kind regards,

Reza Eshraghi, M.D

Academic Editor

PLOS One

Reviewers' comments:

Reviewer's Responses to Questions

**Comments to the Author**

Reviewer #3: All comments have been addressed

Reviewer #4: All comments have been addressed

**********

2. Is the manuscript technically sound, and do the data support the conclusions?

Reviewer #3: Yes

Reviewer #4: Yes

**********

3. Has the statistical analysis been performed appropriately and rigorously?

Reviewer #3: Yes

Reviewer #4: Yes

**********

4. Have the authors made all data underlying the findings in their manuscript fully available?

The PLOS Data policy

Reviewer #3: No

Reviewer #4: Yes

**********

5. Is the manuscript presented in an intelligible fashion and written in standard English?

Reviewer #3: Yes

Reviewer #4: Yes

**********

Reviewer #3: Well done

Thank you all for your tremendous effort, careful revisions, and dedication throughout this process. I believe this current version is robust and acceptable to move forward to the next steps.

Good luck

Reviewer #4: Dear authors,

Thank you for your special attention to the recommended comments.

I have no further comments.

Best wishes

**********

what does this mean?). If published, this will include your full peer review and any attached files.

If you choose “no”, your identity will remain anonymous but your review may still be made public.

**Do you want your identity to be public for this peer review?**  For information about this choice, including consent withdrawal, please see our Privacy Policy

Reviewer #3: No

Reviewer #4: No

**********

# Editor report: Acceptance letter

**DOI:** 10.1371/journal.pone.0360414.r004

**Authors:** Reza Eshraghi

PONE-D-26-25288R1

PLOS One

Dear Dr. Klimczak-Tomaniak,

I'm pleased to inform you that your manuscript has been deemed suitable for publication in PLOS One. Congratulations! Your manuscript is now being handed over to our production team.

At this stage, our production department will prepare your paper for publication. This includes ensuring the following:

* All references, tables, and figures are properly cited

* All relevant supporting information is included in the manuscript submission,

* There are no issues that prevent the paper from being properly typeset

You will receive further instructions from the production team, including instructions on how to review your proof when it is ready. Please keep in mind that we are working through a large volume of accepted articles, so please give us a few days to review your paper and let you know the next and final steps.

Lastly, if your institution or institutions have a press office, please let them know about your upcoming paper now to help maximize its impact. If they'll be preparing press materials, please inform our press team within the next 48 hours. Your manuscript will remain under strict press embargo until 2 pm Eastern Time on the date of publication. For more information, please contact onepress@plos.org.

You will receive an invoice from PLOS for your publication fee after your manuscript has reached the completed accept phase. If you receive an email requesting payment before acceptance or for any other service, this may be a phishing scheme. Learn how to identify phishing emails and protect your accounts at https://explore.plos.org/phishing.

If we can help with anything else, please email us at customercare@plos.org.

Thank you for submitting your work to PLOS One and supporting open access.

Kind regards,

PLOS One Editorial Office Staff

on behalf of

Dr. Reza Eshraghi

Academic Editor

PLOS One
